# Sexual dimorphism in glucose metabolism is shaped by androgen-driven gut microbiome

**DOI:** 10.1038/s41467-021-27187-7

**Published:** 2021-12-06

**Authors:** Aibo Gao, Junlei Su, Ruixin Liu, Shaoqian Zhao, Wen Li, Xiaoqiang Xu, Danjie Li, Juan Shi, Bin Gu, Juan Zhang, Qi Li, Xiaolin Wang, Yifei Zhang, Yu Xu, Jieli Lu, Guang Ning, Jie Hong, Yufang Bi, Weiqiong Gu, Jiqiu Wang, Weiqing Wang

**Affiliations:** 1 Department of Endocrine and Metabolic Diseases, Shanghai Institute of Endocrine and Metabolic Diseases, Ruijin Hospital, Shanghai Jiao Tong University School of Medicine, Shanghai, 200025 China; 2 Shanghai National Clinical Research Center for metabolic Diseases, Key Laboratory for Endocrine and Metabolic Diseases of the National Health Commission of the PR China, Shanghai National Center for Translational Medicine, Ruijin Hospital, Shanghai Jiao Tong University School of Medicine, Shanghai, 200025 China; 3 Aimigene Institute, Shenzhen, 518000 China; 4 CAS Key Laboratory of Separation Science for Analytical Chemistry, Dalian Institute of Chemical Physics, Chinese Academy of Sciences, Dalian, 116023 China

**Keywords:** Metabolic disorders, Endocrine system and metabolic diseases, Microbiome

## Abstract

Males are generally more susceptible to impaired glucose metabolism and type 2 diabetes (T2D) than females. However, the underlying mechanisms remain to be determined. Here, we revealed that gut microbiome depletion abolished sexual dimorphism in glucose metabolism. The transfer of male donor microbiota into antibiotics-treated female mice led the recipients to be more insulin resistant. Depleting androgen via castration changed the gut microbiome of male mice to be more similar to that of females and improved glucose metabolism, while reintroducing dihydrotestosterone (DHT) reversed these alterations. More importantly, the effects of androgen on glucose metabolism were largely abolished when the gut microbiome was depleted. Next, we demonstrated that androgen modulated circulating glutamine and glutamine/glutamate (Gln/Glu) ratio partially depending on the gut microbiome, and glutamine supplementation increases insulin sensitivity in vitro. Our study identifies the effects of androgen in deteriorating glucose homeostasis partially by modulating the gut microbiome and circulating glutamine and Gln/Glu ratio, thereby contributing to the difference in glucose metabolism between the two sexes.

## Abstract (web summary)

Male sex is a risk factor for impaired glucose metabolism and type 2 diabetes. Here the authors identify that androgen modulates the gut microbiome, which drives insulin resistance and contributes to sexual dimorphism in glucose metabolism in mice.

## Introduction

In recent decades, the prevalence of type 2 diabetes (T2D) has rapidly increased worldwide, becoming a global health issue^1^. Of note, population studies have reported a higher prevalence of T2D in men than in women^2,3^. Consistent with humans, male mice have lower insulin sensitivity than female mice^4^ and are more susceptible to high-fat diet (HFD) induced obesity and metabolic syndrome^5^. The sex-dependent glucose metabolism difference is commonly observed in both humans^3,6,7^ and rodent animals^4,8^. However, the underlying mechanisms that drive sexual dimorphism in metabolism remain unclear.

The gut microbiome is a complex microbial ecosystem that engages in nutrient acquisition and energy metabolism, regulating the metabolic health of the host^9^. In recent years, accumulating evidence, including the research of others and our previous studies, has indicated the significant roles of the gut microbiome in metabolic diseases^10–13^. Both Chinese and European population studies have uncovered gut microbiome dysbiosis in T2D^14,15^. The gut microbiome is linked to amino acids, such as glutamine, glutamine/glutamate (Gln/Glu) ratio, and branch-chain amino acids (BCAAs), which affect insulin resistance or metabolic diseases^10–12,16^. Furthermore, the causal relationship between the gut microbiome and insulin resistance or metabolic diseases is indicated by the intestinal microbiota transplantation in mice and bidirectional Mendelian randomization in humans^13,17^.

Interestingly, the gut microbiome is also reported to show different compositions between the sex in both humans and rodent animals^18,19^. A number of bacterial species have been reported to differ in abundance between male and female microbiomes^20^. Moreover, the sex differences in gut microbiome contribute to the sex bias in many physiological and pathological states such as autoimmunity and type 1 diabetes^19,21–23^. However, which factors trigger sexually dimorphic gut microbiome and whether and how this sex difference in the gut microbiome drives sex-biased glucose metabolism remain poorly understood.

Here, we demonstrated that androgen-regulated gut microbiome drives the sex differences in glucose metabolism. Moreover, androgen modulates the circulating glutamine levels and Gln/Glu ratio partially via the gut microbiome, which further affected insulin sensitivity. Thus, we demonstrated that the sex bias of the gut microbiome and glutamine, regulated by androgen, drives the sexual dimorphism in glucose metabolism, revealing previously undetermined links between hormones, the gut microbiome, and amino acids in sex-specific glucose metabolism.

## Results

### Gut microbiome depletion abolishes sex-biased glucose metabolism in normal chow diet (NCD) mice

**Fig. 1:**
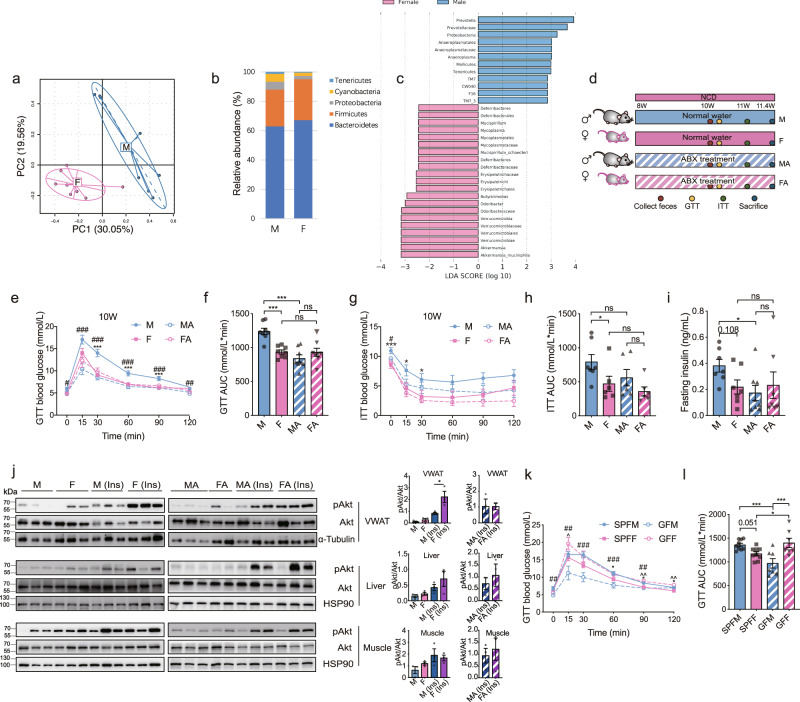
Gut microbiome depletion abolishes sex-biased glucose metabolism in mice fed normal chow diet (NCD). **a** Principal component analysis (PCA) of the gut microbiome in male and female mice based on the operational taxonomic units (OTUs) (mice, *n* = 8 per group). **b** The relative abundances of the five most abundant microbial phyla in male and female mice (mice, *n* = 8 per group). **c** Linear discriminant analysis (LDA) of the gut microbiome between male and female mice. The LDA are used to show the taxa that significantly increased or decreased in the gut microbiota between groups, dominating the uniqueness of the corresponding groups. Scores are used to estimate the contribution of the abundance of each taxon to the differences between the groups. For the positive LDA score in log_10_ in blue bar presents the genera enriched in male mice, while the negative LDA score in log_10_ in red bar presents genera enriched in female mice. Taxa with an LDA score (log_10_) of > 2.0 or < −2.0 are shown (mice, *n* = 8 per group). **d** Schematic diagram of male and female mice with or without antibiotics (ABX) treatment. **e**, **f** Glucose tolerance test (GTT) (**e**) (for M VS MA, *P* = 0.049 at 0 min; for M VS MA, *P* = 5.4 × 10^−4^ at 15 min; for M VS F, *P* = 1.6 × 10^−5^ and for M VS MA, *P* = 3.8 × 10^−6^ at 30 min; for M VS F, *P* = 5.6 × 10^−4^ and for M VS MA, *P* = 7.4 × 10^−5^ at 60 min; for M VS F, *P* = 1.2 × 10^−4^ and for M VS MA, *P* = 1.0 × 10^−4^ at 90 min; for M VS MA, *P* = 0.002 at 120 min) and the corresponding area under the curve (AUC) (**f**) (for M VS F, *P* = 1.2 × 10^−4^ and for M VS MA, *P* = 2.6 × 10^−6^) of the indicated groups (mice, *n* = 8 per group). **g**, **h** Insulin tolerance test (ITT) (**g**) (for M VS F, *P* = 7.9 × 10^−4^ and for M VS MA, *P* = 0.048 at 0 min; for M VS F, *P* = 0.021 at 15 min; for M VS F, *P* = 0.034 at 30 min; for M VS F, *P* = 0.077 at 60 min; for M VS F, *P* = 0.053 at 90 min) and the corresponding AUC (**h**) (for M VS F, *P* = 0.038) of the indicated groups (mice, *n* = 7 for M; *n* = 6 for F, MA, and FA). For **e** and **g**, M vs F, *P < 0.05, **P < 0.01, ***P < 0.001; M vs MA, ^#^*P* < 0.05, ^##^*P* < 0.01, ^###^*P* < 0.001. **i** Six-h fasting plasma insulin levels (for M VS MA, *P* = 0.038) (mice, *n* = 7 for M and F, *n* = 8 for MA; *n* = 7 for FA). **j** Left, representative western blotting of total and phosphorylated Akt (pAkt) levels in visceral white adipose tissues (VWAT), the liver, and muscles of the indicated groups with or without 1 IU/kg insulin stimulation for 10 min. α-Tubulin and HSP90 were used as internal controls (mice, *n* = 2 for FA; *n* = 3 for the other groups). Right, quantification of the intensities of pAkt/Akt corresponding to the western blotting bands (in VWAT, for M ins VS F ins, *P* = 0.044). **k**, **l** GTT (k) (for SPFM VS GFM, *P* = 0.002 at 0 min; for SPFM VS GFM, *P* = 0.009, for SPFF VS GFF, *P* = 0.048, and for GFM VS GFF, *P* = 2.9 × 10^−4^ at 15 min; for SPFM VS GFM, *P* = 3.1 × 10^−4^ and for GFM VS GFF, *P* = 0.001 at 30 min; for SPFM VS SPFF, *P* = 0.039, for SPFM VS GFM, *P* = 5.2 × 10^−4^, and for GFM VS GFF, *P* = 0.002 at 60 min; for SPFM VS SPFF, *P* = 0.022, for SPFM VS GFM, *P* = 0.008, for SPFF VS GFF, *P* = 0.004, and for GFM VS GFF, *P* = 0.001 at 90 min; for SPFM VS SPFF, *P* = 0.039 and for SPFF VS GFF, *P* = 0.001 at 120 min) and the corresponding AUC (**l**) (for SPFM VS SPFF, *P* = 0.051; for SPFM VS GFM, *P* = 4.6 × 10^−4^; for SPFF VS GFF, *P* = 0.026; for GFM VS GFF, *P* = 2.8 × 10^−4^) were detected in specific pathogen-free (SPF) and germ free (GF) mice (mice, *n* = 9 for SPFM and SPFF; *n* = 7 for GFF and GFM). For **k**, SPFM vs SPFF, *P < 0.05; SPFM vs GFM, ^##^*P* < 0.01, ^###^*P* < 0.001; SPFF vs GFF, ^*P* < 0.05, ^^*P* < 0.01. M, male; F, female; MA, male mice after ABX treatment; FA, female mice after ABX treatment. SPFM, male specific pathogen-free mice; SPFF, female specific pathogen-free mice; GFM, male germ-free mice; GFF, female germ-free mice; Ins, insulin. One-way analysis of variance (ANOVA) and the post hoc test of least-significant difference (LSD) (two-sided) were applied to analyze the data in Fig. 1 **e**–**i** and **k**–**l**. Unpaired Student’s *t*-test (two-sided) was performed for the quantitative analysis of western blotting Fig. 1. **j**. Data are expressed as mean ± SEM; *P < 0.05, **P < 0.01, ***P < 0.001; ns, not significant. Source data are provided as a Source Data file.

We firstly compared the gut microbiome between male and female adult mice fed a normal chow diet (NCD) using 16S rRNA gene sequencing. The two groups showed distinct gut microbiome compositions, as demonstrated using principal component analysis (PCA) (Fig. 1a). Bacteroidetes and Firmicutes were the most abundant phyla in all samples (Fig. 1b). Of note, the abundance of *Akkermansia muciniphila* which was reported to have antidiabetic effects^24–26^, was lower in male mice than in females, while the abundance of *Prevotella* was higher in male mice (Fig. 1c). These data demonstrated that the gut microbiome of male mice differed from that of female mice. To test whether the sex differences in the gut microbiome contributed to sex-biased glucose metabolism, we deleted gut microbiome in male and female mice with two weeks of treatment using an antibiotics (ABX) cocktail (Fig. 1d and Supplementary Fig. 1a, b). Male mice presented a poorer glucose tolerance than female mice, while this difference was largely abolished after ABX treatment (Fig. 1e, f). We further detected an interaction effect between the ABX treatment and sex on area under the curve (AUC) of glucose tolerance test (GTT) (*P* = 0.003) (Supplementary Data 1), indicating that male and female mice responded differently to the depletion of the gut microbiome. Insulin tolerance test (ITT) further showed that male mice had lower insulin sensitivity than female mice, while this difference was not completely abolished after ABX treatment (Fig. 1g–i). We did not detect a significant interaction effect between the ABX treatment and sex on insulin sensitivity or fasting insulin levels (Supplementary Data 1). Further, the insulin signaling activities represented by phospho-Akt (pAkt) levels were lower in visceral white adipose tissues (VWAT) of male mice than in females but displayed comparable levels between the two sexes after ABX treatment (Fig. 1j). Notably, the pAkt levels in VWAT of female mice decreased after ABX treatment, which was not parallel to the changes in whole-body insulin sensitivity in ITT in response to ABX treatment (Fig. 1j). This may suggest that other insulin-sensitive tissues that may respond differently to the depletion of gut microbiota by ABX treatment, could also contribute to the whole-body insulin sensitivity, while the underlying mechanisms still need fine explorations. These results suggest that the gut microbiome depletion largely ablates the sexual dimorphism in glucose metabolism. Similarly, fasting plasma triglyceride (TG) levels were higher in male mice than in female mice and this difference was not significant after ABX treatment (Supplementary Fig. 1c). Other metabolic parameters such as total cholesterol (TC), leptin and food intake showed higher trends and body weight was significantly higher in males than in females, while these differences were still present after ABX treatment (Supplementary Fig. 1d–g). No significant interaction effects between the ABX treatment and sex on these parameters (Supplementary Data 1). These data suggest that the trends of higher food intake in males or the difference in body weight between sexes may have limited contribution to the gut microbiome-induced sexual dimorphism in glucose metabolism. In addition, the fasting plasma free fatty acids (FFAs), adiponectin, gut hormones such as glucagon-like peptide-1 (GLP-1), peptide tyrosine tyrosine (PYY) and glucose-dependent insulinotropic polypeptide (GIP) levels, as well as circulating inflammatory factors, including lipopolysaccharides-binding protein (LBP), interleukin-1b (IL-1b), interleukin-6 (IL-6), monocyte chemotactic protein 1 (MCP1), and tumor necrosis factor alpha (TNFα) were also detected, but were not significantly different between the two sexes (Supplementary Fig. 1h–n). Thus, they were not likely to contribute to the sex difference in glucose metabolism. Male and female germ-free (GF) mice were also examined, and male GF mice presented better glucose tolerance than conventionally raised male mice, while female GF mice showed no improvement compared with conventional female mice (Fig. 1k, l). We also detected an interaction effect between GF status and sex on glucose tolerance (*P* for interaction = 0.0001). Again, these results suggest that the glucose tolerance is more affected by the gut microbiome in male mice than in female mice. Additionally, the body weight and food intake remained different in male and female GF mice (Supplementary Fig. 1o, p). Together, these results demonstrate that gut microbiome is required for the sex dimorphism in glucose metabolism.

### Gut microbiome depletion abolishes sex-biased glucose metabolism in high-fat diet (HFD) mice

**Fig. 2:**
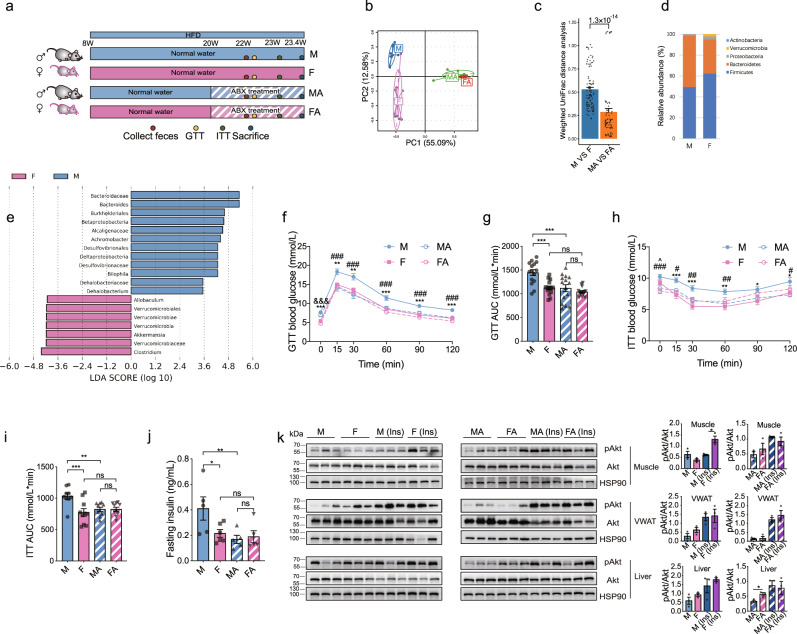
Gut microbiome depletion abolishes sex-biased glucose metabolism in mice fed high-fat diet (HFD). **a** Schematic diagram of male and female mice with or without ABX treatment. **b** PCA of the gut microbiome based on the OTUs of the indicated groups (mice, *n* = 10 for M and F; *n* = 9 for MA; *n* = 8 for FA). **c** Weighted UniFrac distance analysis of the gut microbiome in the indicated groups (mice, *n* = 10 for M and F; *n* = 9 for MA; *n* = 8 for FA). **d** The relative abundance of the five most abundant microbial phyla in male and female mice (mice, *n* = 10 for M and F; *n* = 9 for MA; *n* = 8 for FA). **e** LDA of the gut microbiome between male and female mice. LDA scores were used to estimate the contribution of the abundance of each taxon to the differences between the groups. For the positive LDA score in log_10_ in blue bar presents the genera enriched in male mice, while the negative LDA score in log_10_ in red bar presents genera enriched in female mice. Taxa with an LDA score (log_10_) >2.0 or <−2.0 are shown (mice, *n* = 10 for M and F; *n* = 9 for MA; *n* = 8 for FA). **f**, **g** GTT (**f**) (for M VS F, *P* = 2.2 × 10^−5^ and for MA VS FA, *P* = 3.3 × 10^−5^ at 0 min; for M VS F, *P* = 0.001 and for M VS MA, *P* = 1.3 × 10^−4^ at 15 min; for M VS F, *P* = 0.001 and for M VS MA, *P* = 2.8 × 10^−5^ at 30 min; for M VS F, *P* = 9.5 × 10^−5^ and for M VS MA, *P* = 2.4 × 10^−4^ at 60 min; for M VS F, *P* = 7.2 × 10^−5^ and for M VS MA, *P* = 5.6 × 10^−4^ at 90 min; for M VS F, *P* = 5.6 × 10^−6^ and M VS MA, *P* = 1.9 × 10^−5^ at 120 min) and the corresponding AUC (**g**) (for M VS F, *P* = 8.3 × 10^−5^ and M VS MA, *P* = 5.9 × 10^−5^) of the indicated groups (mice, *n* = 15 for M, F, and MA; *n* = 14 for FA). **h**, **i** ITT (**h**) (for M VS F, *P* = 0.068, for M VS MA, *P* = 9.7 × 10^−4^, and for F VS FA, *P* = 0.014 at 0 min; M VS F, *P* = 4.3 × 10^−4^ and for M VS MA, *P* = 0.014 at 15 min; for M VS F, *P* = 1.5 × 10^−4^ and for M VS MA, *P* = 0.009 at 30 min; for M VS F, *P* = 0.002 and for M VS MA, *P* = 0.009 at 60 min; for M VS F, *P* = 0.017 at 90 min; for M VS F, *P* = 0.032 and for M VS MA, *P* = 0.012 at 120 min) and the corresponding AUC (**i**) (for M VS F, *P* = 3.2 × 10^−4^ and for M VS MA, *P* = 0.003) of the indicated groups (mice, *n* = 9 for M; *n* = 10 for F, MA, and FA). For **f** and **h**, M vs F, *P < 0.05, **P < 0.01, ***P < 0.001; MA vs FA, ^&&&^*P* < 0.001; M vs MA, ^#^*P* < 0.05, ^##^*P* < 0.01, ^###^*P* < 0.001; F vs FA, ^*P* < 0.05. **j** Six-h fasting plasma insulin levels (for M VS F, *P* = 0.018 and for M VS MA, *P* = 0.006) (mice, *n* = 5 for M, *n* = 6 for F; *n* = 5 for MA and FA). **k** Left, representative western blotting of total and pAkt in muscles, VWAT, and the liver of the indicated groups with or without 1 IU/kg insulin stimulation for 10 min. HSP90 was used as an internal control (mice, *n* = 3 per group). Right, quantification of the intensities of pAkt/Akt corresponding to the western blotting bands (in muscle, for M ins VS F ins, *P* = 0.010 and in liver, for MA VS FA, *P* = 0.025). LDA, linear discriminant analysis; ABX, antibiotics; GTT, glucose tolerance test; AUC, area under the curve; ITT, Insulin tolerance test; VWAT, visceral white adipose tissues. M, male; F, female; MA, male mice after ABX treatment; FA, female mice after ABX treatment. Ins, insulin. Data in Fig. 2**c** were tested by two-tailed Wilcoxon rank-sum test. One-way ANOVA and the post hoc test of LSD (two-sided) were applied to analyze the data in Fig. 2**f**–**j**. Unpaired Student’s t-test (two-sided) was performed for the quantitative analysis of western blotting in Fig. 2**k**. Data are expressed as mean ± SEM; *P < 0.05, **P < 0.01, ***P < 0.001; ns, not significant. Source data are provided as a Source Data file.

A HFD consumption is considered as a major environmental risk factor for metabolic diseases. We next examined the gut microbiome and metabolic changes in mice challenged with HFD with or without ABX treatment (Fig. 2a). Consistently, the gut microbiome of HFD male mice was distinguished from that of female mice and was similar after ABX treatment (Fig. 2b, c). Firmicutes and Bacteroidetes were the most abundant phyla in both sexes (Fig. 2d). *Akkermansia* was less enriched in male HFD mice than in females (Fig. 2e), which was consistent with the results in NCD mice. While, *Prevotella* was not identified as the dominant genus in males. Furthermore, glucose tolerance and insulin sensitivity were impaired and the fasting insulin levels were higher in male HFD mice than in females, which were improved to the comparable levels to those of female mice after ABX treatment (Fig. 2f–j). We further detected significant interaction effects of ABX treatment and sex on AUCs of GTT and ITT in HFD mice (*P* value = 0.028 and 0.007, respectively) (Supplementary Data 1), which indicated different responses of the two sexes to the depletion of the gut microbiome in glucose tolerance and insulin sensitivity in mice challenged with HFD. Moreover, ABX-erased sex-difference in glucose metabolism was mainly due to the improvement of male mice in response to gut microbiome depletion (Fig. 2f–j). Further, the pAkt levels in the muscles were lower in male mice compared to females, and this difference was abolished after the ABX treatment (Fig. 2k). In addition, metabolic factors such as plasma levels of FFA and adiponectin levels also differed between male and female mice and were comparable after ABX treatment (Supplementary Fig. 2a, b). While the higher plasma TG and leptin levels in men were not completely abolished by ABX treatment (Supplementary Fig. 2c, d). Moreover, other metabolic parameters such as TC, body weight and food intake were higher in male mice than in females; however, these metabolic differences remained significant after ABX treatment (Supplementary Fig. 2e-g). No interaction effects were detected between ABX treatment and sex on these parameters (Supplementary Data 1), suggesting that these metabolic differences between sexes did not depend on the gut microbiome. Gut hormones, including active GLP-1, PYY, and GIP, were also examined; however, no significant difference was observed between the two sexes either before or after ABX treatment (Supplementary Fig. 2h-j). These data consistently demonstrated that the gut microbiome was involved in the sex difference in glucose metabolism in mice challenged with HFD.

### Sex-specific gut microbiome drives sexual dimorphism in insulin sensitivity

**Fig. 3:**
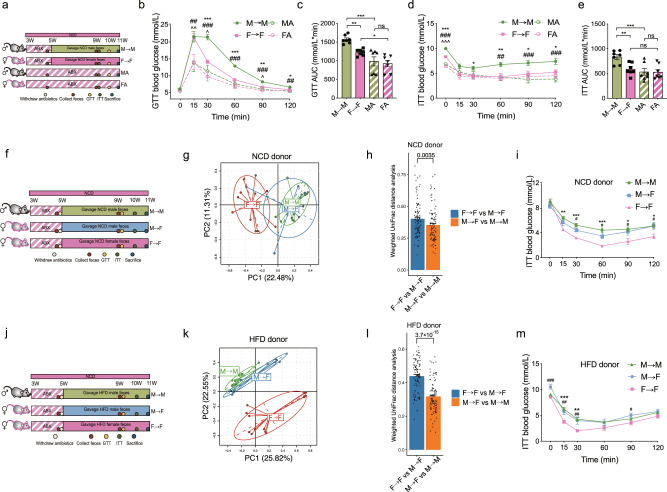
Sex-specific gut microbiome drives sexual dimorphism in insulin sensitivity. **a** Schematic diagram of the gut microbiome reconstruction in male and female recipient mice receiving feces from NCD-fed male and female donor mice respectively (mice, *n* = 7 per group). **b**, **c** GTT (**b**) (for M → M VS MA, *P* = 0.006 and for F → F VS FA, *P* = 0.009 at 15 min; for M → M VS F → F, *P* = 3.8 × 10^−4^, for M → M VS MA, *P* = 5.9 × 10^−6^, and F → F VS FA, *P* = 0.017 at 30 min; for M → M VS F → F, *P* = 1.4 × 10^−4^ and for M → M VS MA, *P* = 5.1 × 10^−6^ at 60 min; for M → M VS F → F, *P* = 0.002, for M → M VS MA, *P* = 7.1 × 10^−5^, and for F → F VS FA, *P* = 0.038 at 90 min; for M → M VS F → F, *P* = 0.041 and for M → M VS MA, *P* = 0.002 at 120 min) and the corresponding AUC (**c**) (for M → M VS F → F, *P* = 0.005, for M → M VS MA, *P* = 2.0 × 10^−5^, and for F → F VS FA, *P* = 0.012) in the indicated groups (mice, n = 7 per group). **d**, **e** ITT (d) (for M → M VS F → F, *P* = 1.5 × 10^−6^, for M → M VS MA, *P* = 1.1 × 10^−11^, and for F → F VS FA, *P* = 1.3 × 10^−7^ at 0 min; for M → M VS F → F, *P* = 0.041 at 30 min; for M → M VS F → F, *P* = 0.004 and for M → M VS MA, *P* = 0.003 at 60 min; for M → M VS F → F, *P* = 0.013 and for M → M VS MA, *P* = 5.6 × 10^−4^ at 90 min; for M → M VS F → F, *P* = 0.012 and for M → M VS MA, *P* = 1.6 × 10^−4^ at 120 min) and the corresponding AUC (**e**) (for M → M VS F → F, *P* = 0.004 and for M → M VS MA, *P* = 7.0 × 10^−4^) in the indicated groups (mice, *n* = 7 per group). **f** Schematic diagram of FMT in female recipient mice receiving feces from NCD-fed male and female donor mice respectively. **g** PCA of the gut microbiome based on the OTUs of the indicated groups (mice, *n* = 11 for M → M and F → F; *n* = 9 for M → F). The donors were fed the NCD. **h** Weighted UniFrac distance analysis of the gut microbiome in the indicated groups (mice, *n* = 11 for M → M and F → F; *n* = 9 for M → F). The donors were fed a NCD. **i** ITT in M → M, M → F, and F → F groups (for M → M VS F → F, *P* = 0.004 at 15 min; for M → M VS F → F, *P* = 3.1 × 10^−4^ and for M → F VS F → F, *P* = 0.019 at 30 min; for M → M VS F → F, *P* = 6.7 × 10^−4^ and for M → F VS F → F, *P* = 0.020 at 60 min; for M → M VS F → F, *P* = 0.015 and for M → F VS F → F, *P* = 0.045 at 90 min; for M → M VS F → F, *P* = 0.033 and for M → F VS F → F, *P* = 0.032 at 120 min) (mice, *n* = 11 for M → M; *n* = 7 for F → F; *n* = 11 for M → F). The donors were fed a NCD. **j** Schematic diagram of FMT in female recipient mice receiving feces from HFD-fed male and female donor mice respectively. **k** PCA of the gut microbiome based on the OTUs of the indicated groups (mice, *n* = 13 for M → M and F → F; *n* = 8 for M → F). The donors were fed a HFD. **l** Weighted UniFrac distance analysis of the gut microbiome in the indicated groups (mice, *n* = 13 for M → M and F → F; *n* = 8 for M → F). The donors were fed a HFD. **m** ITT in M → F, M → F, and F → F groups (for M → F VS F → F, *P* = 3.8 × 10^−4^ at 0 min; for M → M VS F → F, *P* = 1.7 × 10^−4^ and for M → F VS F → F, *P* = 0.002 at 15 min; for M → M VS F → F, *P* = 0.002 and for M → F VS F → F, *P* = 0.006 at 30 min; for M → F VS F → F, *P* = 0.012 at 90 min) (mice, *n* = 13 for M → M; *n* = 12 for F → F and M → F). The donors were fed a HFD. ABX, antibiotics; GTT, glucose tolerance test; AUC, area under the curve; ITT, Insulin tolerance test; NCD, normal chow diet; HFD, high fat diet. M → M, male feces transferred to male recipients; F → F, female feces transferred to female recipients; MA, male mice with ABX treatment; FA, female mice with ABX treatment. M → F, male feces transferred to female recipients. For Fig. 3**b**, **d**, M → M VS F → F, *P < 0.05, **P < 0.01, ***P < 0.001; M → M VS MA, ^#^*P* < 0.05, ^##^*P* < 0.01, ^###^*P* < 0.001; F → F VS FA, ^^^*P* < 0.05, ^^^^ *P* < 0.01, ^^^^^ *P* < 0.001. For Fig. 3**i**, **m**, M → M VS F → F, *P < 0.05, **P < 0.01, ***P < 0.001; M → F VS F → F, ^#^*P* < 0.05, ^##^*P* < 0.01, ^###^*P* < 0.001. One-way ANOVA and the post hoc test of LSD (two-sided) were applied to analyze the data in Fig. 3**b**–**e**, **i**, and **m**. Data in Fig. 3**h** and **l** were tested by two-tailed Wilcoxon rank-sum test. Data are expressed as mean ± SEM. Source data are provided as a Source Data file.

To identify the effects of the sex-specific gut microbiome on glucose homeostasis, we further conducted a fecal microbiota transplantation (FMT) experiment, includuing four groups of male and female mice. After treatment with ABX for two weeks, one group of male mice were gavaged with feces from male donors, and one group of female mice were gavaged with feces from female donors to recapitulate the sex differences in the gut microbiome. While the other two groups of male and female mice maintained the ABX treatment without FMT as controls. ITT and GTT were performed after the mice being gavaged for another four weeks (Fig. 3a). Consequently, male and female mice did not show significant differences in glucose tolerance and insulin sensitivity with ABX treatment, while the two groups with FMT resumed the sex differences in glucose tolerance and insulin sensitivity (Fig. 3b–e). Notably, we observed a significant interactive effect between sex and the gut microbiome reconstruction on blood glucose levels at 30 and 60 min after glucose injection in GTT (Supplementary Data 1). Meanwhile, this interactive effect was also observed on blood glucose levels at 0, 90, and 120 min after insulin injection as well as for the AUC of ITT (Supplementary Data 1). These results demonstrated the driving role of the gut microbiome in the sexual dimorphism in glucose metabolism.

To further verify the causal effects of the gut microbiome on the sex difference in glucose metabolism, We transferred male and female donor feces to antibiotics-treated female recipients (M → F vs F → F) at five weeks, respectively, and also transferred male donor feces to antibiotics-treated male recipients (M → M) (Fig. 3f). As expected, when gavaged with the feces from NCD-fed donors, the gut microbiome of the M → F group was distinct from the F → F group and was more closed to the M → M group, as reflected by the PCA analysis and weighed UniFrac distance (Fig. 3g, h and Supplementary Fig. 3a). More importantly, female recipient mice receiving male donor feces (M → F group) also showed reduced insulin sensitivity than the F → F group (Fig. 3i). While, glucose tolerance was not different between two groups (Supplementary Fig. 3b). In consistence, when gavaged with the feces from HFD-fed donors (Fig. 3j), the M → F group showed distinct microbial compositions (Fig. 3k, l and Supplementary Fig. 3c) and impaired insulin sensitivity (Fig. 3m and Supplementary Fig. 3d) compared with those of the F → F group. These results together demonstrated that FMT could reproduce the sexual dimorphism in the gut microbiome and glucose metabolism, and that the male microbiome led to insulin resistance, contributing to sex-biased glucose metabolism.

### Androgen deteriorates glucose metabolism via modulating the gut microbiome

**Fig. 4:**
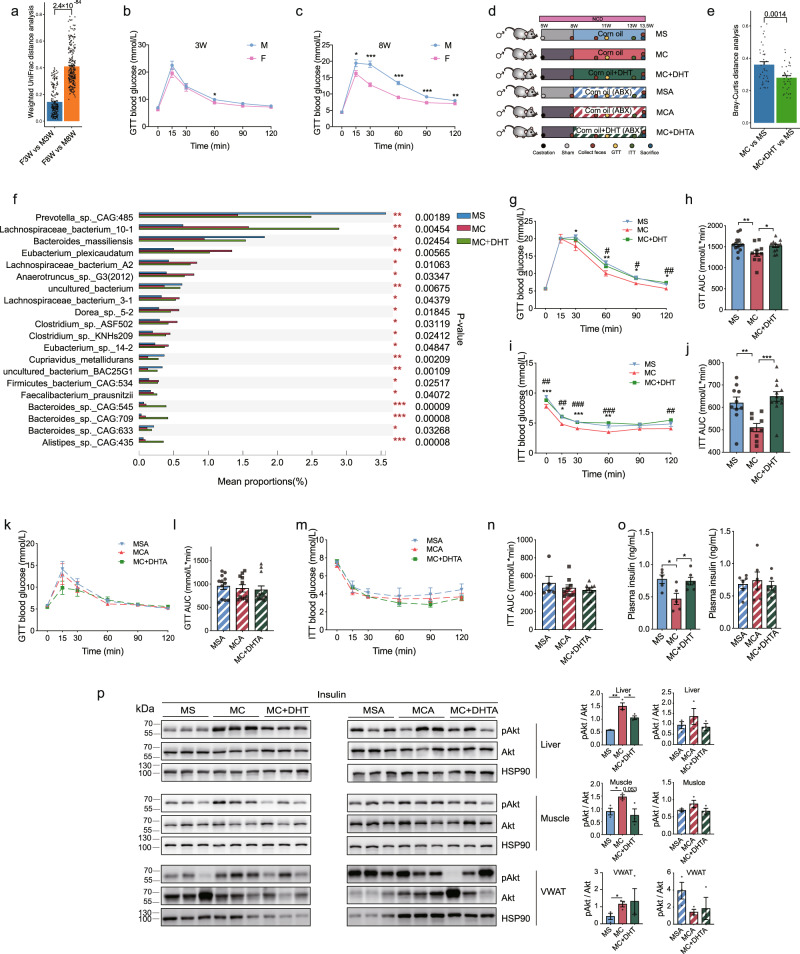
Androgen deteriorates glucose metabolism via modulating the gut microbiome. **a** Weighted UniFrac distance analysis of the gut microbiome in the indicated groups (mice, *n* = 14 for M3W; *n* = 17 for M8W; *n* = 16 for F3W and F8W). **b**, **c** GTT between male and female mice at three weeks (**b**) (for M VS F, *P* = 0.038 at 60 min) and eight weeks old (**c**) (for M VS F, *P* = 0.033 at 15 min; *P* = 7.4 × 10^−6^ at 30 min; *P* = 1.0 × 10^−7^ at 60 min; *P* = 1.0 × 10^−4^ at 90 min; *P* = 0.008 at 120 min) (mice, *n* = 17 for M3W and M8W; *n* = 16 for F3W and F8W). **d** Schematic diagram of male mice subjected to castration and DHT injection without or with ABX treatment. **e** The Bray-Curtis distance analysis among the indicated groups based on the metagenomics data of the gut microbiome (mice, *n* = 5 for MS; *n* = 8 for MC; *n* = 8 for MC + DHT). **f** The top 20 abundant species with differences in abundance in MS, MC and, MC + DHT groups (mice, *n* = 5 for MS; *n* = 8 for MC; *n* = 8 for MC + DHT). Taxonomic annotations were against NCBI NR database and one-way ANOVA was used for the abundance comparison in the groups. **g**, **h** GTT (**g**) (for MS VS MC, *P* = 0.035 at 30 min; for MS VS MC, *P* = 0.005 and for MC VS MC + DHT, *P* = 0.039 at 60 min; for MS VS MC, *P* = 0.011 and for MC VS MC + DHT, *P* = 0.022 at 90 min; for MS VS MC, *P* = 0.028 and for MC VS MC + DHT, *P* = 0.002 at 120 min) and the corresponding AUC (h) (for MS VS MC, *P* = 0.009 and for MC VS MC + DHT, *P* = 0.034) in the indicated groups (mice, *n* = 13 for MS; *n* = 10 for MC; *n* = 12 for MC + DHT). **i**, **j** ITT (**i**) (for MS VS MC, *P* = 1.1 × 10^−4^ and for MC VS MC + DHT, *P* = 0.005 at 0 min; for MS VS MC, *P* = 0.011 and for MC VS MC + DHT, *P* = 0.003 at 15 min; for MS VS MC, *P* = 5.6 × 10^−5^ and for MC VS MC + DHT, *P* = 2.8 × 10^−5^ at 30 min; for MS VS MC, *P* = 0.008 and for MC VS MC + DHT, *P* = 4.9 × 10^−4^ at 60 min; for MC VS MC + DHT, *P* = 0.004 at 120 min) and the corresponding AUC (**j**) (for MS VS MC, *P* = 0.002 and for MC VS MC + DHT, *P* = 1.5 × 10^−4^) of the indicated groups (mice, *n* = 10 for MS; *n* = 9 for MC; *n* = 12 for MC + DHT). For **g** and **i**, MS vs MC, *P < 0.05, **P < 0.01, ***P < 0.001; MC vs MC + DHT, ^#^*P* < 0.05, ^##^*P* < 0.01, ^###^*P* < 0.001. **k**, **l** GTT (**k**) and the corresponding AUC (**l**) of the indicated groups with ABX treatment (mice, *n* = 12 for MSA; *n* = 11 for MCA; *n* = 12 MC + DHTA). **m**, **n** ITT (**m**) and the corresponding AUC (**n**) of the indicated groups with ABX treatment (mice, *n* = 5 for MSA; *n* = 9 for MCA and MC + DHTA). **o** Six-h fasting plasma insulin levels (for MS VS MC, *P* = 0.010 and for MC VS MC + DHT, *P* = 0.014) (mice, *n* = 5 for MS and MC; *n* = 6 for MCA, MSA, MC + DHT, and MC + DHTA). **p** Left, representative western blotting of total and pAkt in the liver, muscles, and VWAT of the indicated groups with 1 IU/kg insulin stimulation for 10 min. HSP90 was used as an internal control (mice, *n* = 3 for each group). Right, quantification of the intensities of pAkt/Akt corresponding to the western blotting bands (in liver, for MS VS MC, *P* = 0.002 and for MC VS MC + DHT, *P* = 0.041; in muscle, for MS VS MC, *P* = 0.021 and for MC VS MC + DHT, *P* = 0.053; in VWAT, for MS VS MC, *P* = 0.042). F3W, three-week female mice; M3W, three-week male mice; F8W, egitht-week female mice; M8W, eight-week male mice. ABX, antibiotics; GTT, glucose tolerance test; AUC, area under the curve; ITT, insulin tolerance test; VWAT, visceral white adipose tissues. MS, male sham mice; MC, male castrated mice; MC + DHT, male castrated mice injected with DHT. MSA, male sham mice under ABX treatment; MCA, male castrated mice under ABX treatment, MC + DHTA, male castrated mice injected with DHT under ABX treatment. One-way ANOVA and the post hoc test of LSD (two-sided) were applied to analyze the data in Fig. 4**g**-**o**. Unpaired Student’s t-test (two-sided) was performed for Fig. 4**b**–**c** and the quantitative analysis of western blotting in Fig. 4**p**. Data in Fig. 4**a** and **e** were tested by two-tailed Wilcoxon rank-sum test. Data are expressed as mean ± SEM. Source data are provided as a Source Data file.

Sex hormones are reported to regulate gut microbiome in certain states^19,21,27^. We next compared the gut microbiome between male and female mice at weaning (sexually immature) and eight weeks (sexually mature), and found that the weighted UniFrac distance between adult male and female mice was significantly higher than that between the immature two sexes (Fig. 4a). Consistently, glucose tolerance was overall identical between the two sexes at weaning time but it was significantly different at eight weeks of age (Fig. 4b, c). These results suggest that sex hormones may be involved in sex-biased gut microbiome and glucose metabolism. As mentioned above, glucose metabolism was more robustly affected by gut microbiome in male mice than in female mice. We then castrated male mice to investigate the effect of androgen on gut microbiome and glucose metabolism (Supplementary Fig. 4a). Interestingly, the microbiome in castrated male mice had lower distance from females than that between males and females (Supplementary Fig. 4b, c), suggesting that androgen is an important factor driving the difference of gut microbiome between the two sexes. Meanwhile, glucose tolerance and insulin sensitivity were also improved after castration (Supplementary Fig. 4d, e). These results suggest that androgen regulates gut microbiome and glucose metabolism. To further explore whether the effect of androgen on glucose metabolism was dependent on the gut microbiome, we castrated male mice and reintroduced dihydrotestosterone (DHT) with or without ABX treatment (Fig. 4d). The distance between male castrated mice and male sham mice were significantly higher than that between the DHT group and sham males (Fig. 4e), supporting that castration changed the gut microbiome, and DHT treatment drove it similar towards males. Of note, among the differed top 20 abundant species in the three groups, we detected that the abundance of *Prevotella sp. Bacteroides massiliensis and Cupriavidus metallidurans* was decreased while the abundance of *Eubacterium plexicaudatum, Lachnospiraceae bacterium A2 and Dorea_sp*. was increased after castration as compared with sham mice or DHT-treated group (Fig. 4f). Consequently, castration improved glucose tolerance and insulin sensitivity in male mice while DTH treatment deteriorated glucose metabolism (Fig. 4g–j). Importantly, the effects of androgen on glucose metabolism were largely abolished when deleting gut microbiome via ABX treatment (Fig. 4k–n), which was also supported by the detection of the interaction effects of DHT and ABX treatment on glucose levels during GTT or ITT (Supplementary Data 1). Together, these results indicated that the effects of androgen on glucose metabolism were largely dependent on the gut microbiome. Consistent results were observed in fasting plasma insulin levels (Fig. 4o and Supplementary Data 1). Besides, the pAkt levels were increased in the liver and muscles of the castrated male mice as compared with male sham mice but were decreased in the liver and showed decreased trend in muscles after DHT injection (Fig. 4p). These differences were also blunted under ABX treatment. Together, these results demonstrated that androgen deteriorated glucose tolerance and insulin sensitivity, partially via modulation of the gut microbiome, contributing to the sexual dimorphism in glucose metabolism.

### Androgen influences circulating glutamine and Gln/Glu ratio via the gut microbiome

**Fig. 5:**
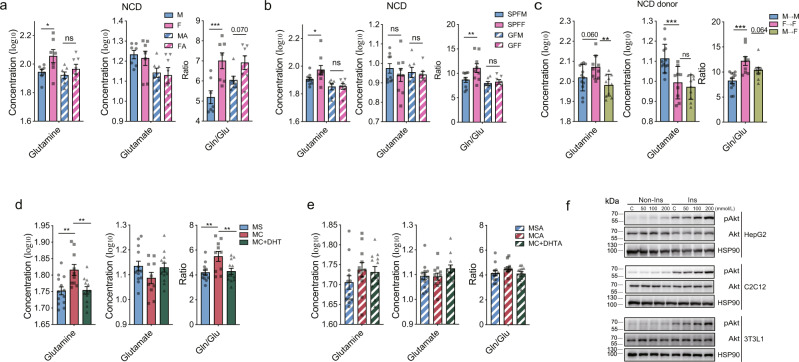
Androgen influences circulating glutamine and glutamine/glutamate (Gln/Glu) ratio via gut microbiome. **a**, Plasma concentrations of glutamine, glutamate, and Gln/Glu ratio in male and female mice fed a NCD without or with ABX treatment (in glutamine, for M VS F, *P* = 0.021; in Gln/Glu, for M VS F, *P* = 5.6 × 10^−4^) (mice, *n* = 7 per group). **b** Plasma concentrations of glutamine, glutamate, and Gln/Glu ratio in male and female SPF and GF mice (in glutamine, for SPFM VS SPFF, *P* = 0.042; in Gln/Glu, for SPFM VS SPFF, *P* = 0.009) (mice, *n* = 9 for SPFM and SPFF; *n* = 7 for GFM and GFF). **c** Plasma concentrations of glutamine, glutamate, and Gln/Glu ratio in the indicated groups in FMT experiment with feces from mouse donor fed a NCD (in glutamine, for M → M VS F → F, *P* = 0.060; for M → F VS F → F, *P* = 0.003; in glutamate, for M → M VS F → F, *P* = 6.7 × 10^−4^; in Gln/Glu, for M → M VS F → F, *P* = 1.8 × 10^−4^ and for M → F VS F → F, *P* = 0.064) (mice, *n* = 12 for M → M; *n* = 9 for F → F, *n* = 10 for M → F). **d**, **e** Plasma concentrations of glutamine, glutamate, and Gln/Glu ratio in the indicated groups in the castration and DHT supplementation experiment without (**d**) (in glutamine, for MS VS MC, *P* = 0.002 and for MC VS MC + DHT, *P* = 0.003; in Gln/Glu, for MS VS MC, *P* = 0.002 and for MC VS MC + DHT, *P* = 0.005) (mice, *n* = 13 for MS; *n* = 10 for MC; *n* = 12 for MC + DHT) or with antibiotics treatment (**e**) (mice, *n* = 12 for MSA, *n* = 11 for MCA; *n* = 12 for MC + DHTA). For **a**–**e**, the concentrations of glutamine and glutamate were calculated as log_10_ and the ratio was calculated directly by the glutamine concentration to the glutamate concentration. **f** Representative western blotting of total and pAkt in the HepG2, differentiated C2C12, and 3T3-L1 cells of the indicated groups without or with 200 nmol/L insulin stimulation for 12 min. HSP90 was used as an internal control. This experiment was repeated independently for three times. M, male; F, female; MA, male mice after ABX treatment; FA, female mice after ABX treatment. SPFM, male specific pathogen free mice; SPFF, female specific pathogen free mice; GFM, male germ-free mice. GFF, female germ-free mice. M → M, male feces transferred to male recipients; F → F, female feces transferred to female recipients; M → F, male feces transferred to female recipients. MS, male sham mice; MC, male castrated mice; MC + DHT, male castrated mice injected with DHT; MSA, male sham mice under ABX treatment; MCA, male castrated mice under ABX treatment, MC + DHTA, male castrated mice injected with DHT under ABX treatment. Ins, insulin. *P < 0.05, **P < 0.01, ***P < 0.001; ns, not significant. One-way ANOVA and the post hoc test of LSD (two-sided) were applied to analyze the data in Fig. 5**a**–**e**. Data are expressed as mean ± SEM. Source data are provided as a Source Data file.

Amino acids are well known metabolites that regulate insulin resistance and the development of T2D^28–30^ and can be metabolized by the gut microbiota^31^. We next compared the blood amino acid profiles between male and female mice with or without ABX treatment. We observed significantly different amino acid profiles in male and female NCD mice (Fig. 5a and Supplementary Fig. 5a–d). Among them, the concentrations of glutamine and the Gln/Glu ratio were lower in male mice than in females but these differences were not significant after ABX treatment or in GF mice (Fig. 5a, b and Supplementary Fig. 5e–g). We also observed a potential interaction effect between the GF status and sex on Gln/Glu ratio (*P* = 0.116) (Supplementary Data 1). As glutamine and the Gln/Glu ratio have been repeatedly reported as protective factors for glucose metabolism and cardiometabolic events^30,32,33^, we further tested their changes in other models. Consistently, plasma glutamine and Gln/Glu ratio were also lower in male mice than in females fed HFD, and these differences were not significant after ABX treatment (Supplementary Fig. 5h and Supplementary Data 1), consistent to the results in NCD mice. Further, in FMT using feces from NCD fed donor mice, plasma glutamine levels and Gln/Glu ratio showed lower trend or significantly lower in the M → M group and the M → F group as compared with F → F group (Fig. 5c). In FMT using feces from HFD fed donor mice, although no difference was seen between the M → F group and the F → F group, plasma levels of glutamine and Gln/Glu ratio were significantly lower in the M → M group than in the F → F group (Supplementary Fig. 5i). These results suggest that the gut microbiome reconstitution partially triggers sex differences in the circulating glutamine levels and the Gln/Glu ratio. To further explore the effects of androgen on glutamine and Gln/Glu ratio and whether these effects were dependent on gut microbiome, we examined the glutamine and glutamate levels in male castrated mice and castrated mice injected with DHT in the presence or absence of antibiotics treatment. Consistently, we observed that castration increased while DHT decreased circulating glutamine levels and Gln/Glu ratio, while these alterations were not significant with ABX treatment (Fig. 5d, e). A borderline interaction effect was detected between castration or DHT treatment with ABX treatment (Supplementary Data 1). These results together suggest that androgen regulates the glutamine levels and the Gln/Glu ratio, which was partially mediated by the gut microbiome. To evaluate the biological roles of glutamine on insulin sensitivity, we next treated HepG2, differentiated C2C12, and 3T3-L1 cells with glutamine in vitro, and found that the pAkt levels were elevated with the increase in glutamine concentrations, demonstrating that glutamine directly improved insulin sensitivity in hepatocytes, myocytes and adipocytes (Fig. 5f). In summary, an androgen-regulated gut microbiome influences the sexual dimorphism in the circulating glutamine levels, further regulating insulin sensitivity.

### Gln/Glu ratio is associated with insulin sensitivity and androgen in humans

**Fig. 6:**
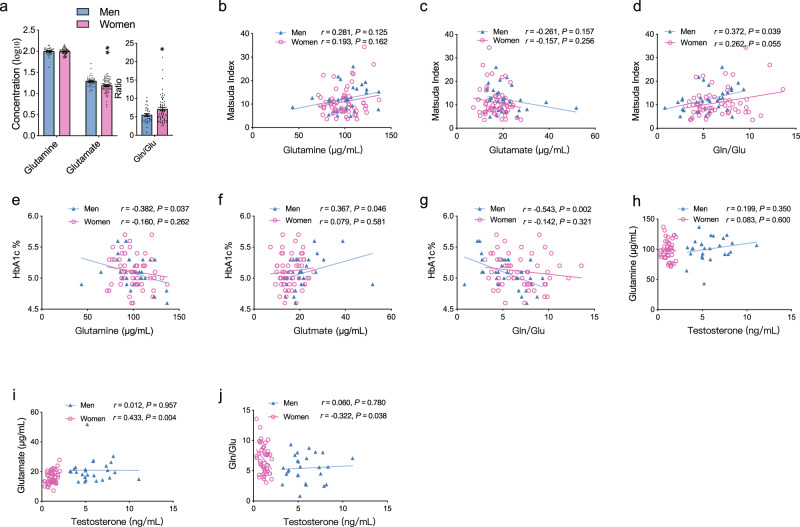
Gln/Glu ratio is associated with insulin sensitivity and androgen in humans. **a** Serum concentrations of glutamine, glutamate, and Gln/Glu ratio in men (*n* = 33) and women (*n* = 63) (in glutamate, for men VS women, *P* = 0.001; in Gln/Glu, for men VS women, *P* = 0.021). The concentrations of glutamine and glutamate were calculated as log_10_ and the ratio was calculated directly by the glutamine concentration to the glutamate concentration. **b**–**j** Partial correlation analysis of circulating glutamine, glutamate and Gln/Glu ratio with Matsuda index (**b**–**d**) (Men, *n* = 33; Women, *n* = 56), HbA1c (**e**–**g**) (Men, *n* = 32; Women, *n* = 53), and serum testosterone levels (**h**–**j**) (Men, *n* = 26; Women, *n* = 44) in men and women, respectively, adjusted for age and BMI. *r* and *P* value were based on the correlation in each sex. Each point represents a single sample. Blue points represent men and red points represent women. Gln/Glu, glutamine/glutamate. *P < 0.05, **P < 0.01. Unpaired Student’s t-test (two-sided) was performed for Fig. 6**a**. Data are expressed as mean ± SEM. Source data are provided as a Source Data file.

We next compared the serum glutamine, glutamate, and the Gln/Glu ratio in healthy young male with those of female individuals, who were recruited in our previous research^12^. Although there was no significant difference in the serum glutamine levels, men showed higher glutamate levels and lower Gln/Glu ratio than women (Fig. 6a), which may suggest that disturbed Gln/Glu transition might exist in men. We then examined the associations of glutamine, glutamate and their ratio with the glucose parameters after adjusting for age and body mass index (BMI). Matsuda index, an indicator for insulin sensitivity, which is correlated with the rate of whole-body glucose disposal during the euglycemic insulin clamp^34^, showed a significant positive correlation with Gln/Glu ratio in men and a correlation trend in women (Fig. 6b–d). HbA1c showed an inverse correlation with glutamine levels and the Gln/Glu ratio, and a positive correlation with glutamate levels in men (Fig. 6e–g). Additionally, the serum testosterone was also measured and was positively correlated with glutamate and negatively correlated with Gln/Glu ratio in women (Fig. 6h–j). Together, these results suggest that lower Gln/Glu ratio correlated with a worsened glucose metabolism especially in men.

## Discussion

Metabolic differences between males and females are commonly observed across various species^35,36^. However, the detailed mechanism remains largely undetermined. Here, we demonstrated the essential role of the gut microbiome especially that of males, in driving sexual difference in glucose metabolism and revealed the role of androgens in regulating the glucose metabolism through the gut microbiome. Finally, we demonstrated that the gut microbiome drives sex bias in circulating glutamine and Gln/Glu ratio, which are associated with glucose metabolism.

We found that male adult mice showed a poorer glucose tolerance and insulin sensitivity, along with having a different gut microbiome composition from that of female adult mice. Of note, *Akkermansia* was remarkably lower in male mice than in female mice. Previous studies have demonstrated the anti-obesity and anti-diabetic effects of *Akkermansia muciniphila* that are associated with a healthy metabolic status^24–26^. While *Prevotella* was higher in males and *Prevotella sp*. were further reduced by castration. These changed microbial species might be the potential species contributing to the sex-biased glucose metabolism and warranted future examinations. Further, we found that the sex difference in glucose metabolism was eliminated when the gut microbiome was depleted by using an ABX cocktail. We observed an interaction effect of ABX treatment and sex on glucose tolerance, and insulin sensitivity especially in mice challenged with HFD. Of note, male mice responded more sensitively to gut microbiome depletion in regulating glucose metabolism. Further, reconstructing male and female gut microbiome via FMT induced the sex difference in glucose metabolism and insulin sensitivity. Moreover, transfer of male donor microbiota led the female recipient mice to be more insulin resistant, indicating the crucial role of the male gut microbiome in driving sex-biased glucose metabolism.

A previous study reported that the composition of the gut microbiome diverges in male and female mice after puberty and is influenced by androgen in non-obese diabetic (NOD) mice^21^. Human studies have also reported a more variable gut microbiota in men, which is strongly correlated with age, and the sex differences in the gut microbiome observed in younger adults are considerably reduced in the elderly population^37^, suggesting the potential involvement of sex hormones in microbial changes. Consistently, we found that the gut microbiome of castrated male mice was more closed to that of females, while reintroducing DHT in the castrated mice largely changed their gut microbiome back towards that of male mice, suggesting that androgen is an important factor in regulating the gut microbiome difference between the sexes. Meanwhile, glucose metabolism was also improved in castrated male mice, and it was deteriorated after DHT treatment. Moreover, the effects of androgen on glucose metabolism were largely eliminated in the absence of the gut microbiome, suggesting that the gut microbiome was required for androgen’s effect on glucose metabolism. Why do men possess a higher blood glucose levels compared with that of women? This may have evolutionary advantages for men to get through the much harder starvation times by maintaining higher blood glucose levels in the primate hunting life, as for the majority of mammals, males afford more physical activities, including hunting and fighting, than females in the wild and primate lifestyle. According to “the host-microbiome evolution theory”, host-level selection shows a higher potential to favor beneficial strains, which may improve the host fitness and survival^38^. However, this advantage may rather become a new challenge with an abundant food supply and a sedentary lifestyle nowadays. Sex-specific effects of gut microbiome are also observed in other physiological or pathological processes such as immunity or type 1 diabetes^19,21^. These findings suggest that androgen might be a common factor in shaping the sex difference in gut microbiome, thereby influencing sex-biased human diseases, such as autoimmune diseases or metabolic diseases. Besides androgen, other factors such as estrogen may also have contributions to sex-biased glucose metabolism, as estrogen was previously reported to protect females from insulin resistance through lowering inflammation levels in adipose tissues and liver, and increasing glucose uptake in muscle in previous reports^39–42^. Estrogen supplementation or ovariectomy was also reported to alter the gut microbiome compositions^43^. However, whether the effects of estrogen on glucose metabolism are dependent on the gut microbiome still need more investigations.

In agreement with our findings, previous studies also reported that castrated male mice showed decreased fasting plasma glucose, insulin, HOMA-IR and increased adiponectin levels, close to those of female mice^4,44^. However, the underlying mechanism remains unclear for decades. Here, our findings suggested the mediated role of the gut microbiome in linking androgen and glucose metabolism. Additionally, the effects of androgen on glucose metabolism may be age-specific, as one strain of androgen receptor knockout (ARKO) mice with C57BL/6 and 129 Sv backgrounds develop accelerated hyperglycemia and insulin resistance with age^45^. Another strain with a C57BL/6N background shows late-onset obesity but intact insulin sensitivity at an old age^46^. Testosterone deficiency has also been reported to be associated with metabolic syndrome, mainly in middle-aged and older men^47^. Based on these researches, the risk roles of androgen in glucose metabolism may be especially in relatively young males. The discrepancy of androgen-related metabolic disorders in different ages may lie in the gut microbiome profile, which also varies along with aging^48^. These findings indicate the importance of age as a cofounder for androgen and gut microbiome in regulating glucose homeostasis. Future studies are warranted to examine whether androgen and its regulated gut microbiome yield different effects on glucose metabolism in old mice.

Our previous study also uncovered the links between gut microbiome and amino acids, such as glutamate, in obesity and metabolic disturbances^12^. Here, we observed that the circulating glutamine levels and Gln/Glu ratio were notably lower in male mice and regulated by androgen and the gut microbiome. Glutamine provides essential intermediates in the tricarboxylic acid cycle and is important for a variety of cellular biological functions^49^. We demonstrated that glutamine treatment directly increased insulin sensitivity in the hepatic, muscle, and adipose cells in vitro. This was consistent with previous findings that L-glutamine treatment reduces postprandial blood glucose in patients with T2D^32^, and attenuates hyperglycemia and insulin resistance in HFD mice and rats^50,51^. Glutamine and Gln/Glu ratio have been reported to be associated with a decreased risk of future diabetes and total mortality^30,33^. We also found that the Gln/Glu ratio was positively correlated with Matsuda index and negatively correlated with HbA1c, supporting its protective role in insulin sensitivity and blood glucose levels especially in young men. Together, these findings may suggest that glutamine and Gln/Glu ratio may be one linking factor between the androgen-regulated gut microbiome and insulin sensitivity. Future studies are needed to elucidate how androgen regulates gut microbiome, glutamine levels and Gln/Glu ratio as well as the detailed mechanisms by which glutamine influences the glucose metabolism in male and female mice.

In conclusion, our study identified the essential role of gut microbiome in triggering sex-biased glucose metabolism and uncovered the effects of androgen in deteriorating glucose homeostasis via modulating the gut microbiome and glutamine levels, at least in part. These findings provide the basis for developing drugs targeting the gut microbiome to combat type 2 diabetes, especially in men, and highlight the importance of stratifying male and female diabetic patients when using drugs that may affect the gut microbiome.

## Methods

### Mice

Specific pathogen-free (SPF) grade male and female C57BL/6 mice were purchased from Linchang Company (Shanghai, China), and were housed in a 12 h dark-light cycle room and allowed access to water and food *ad libitum*. Mice were kept at 22–24 °C, in 50–70% humidity. Mice were fed a normal chow diet (NCD) (SLACOM, P1101F-25) or a high-fat diet (HFD) with 60 kcal % fat (Research Diet, D12492i) from eight weeks old of age. Littermates of the same sex were randomly assigned to the experimental groups. The body weight of NCD mice was measured at the beginning and end of the experiments. The body weight of HFD mice was measured every two weeks. Food intake was recorded continuously for three days in the unit of a cage and was calculated as the average food intake per mouse per day. Male and female eight-week-old germ-free (GF) C57BL/6 mice were imported from Taconic Biosciences (Rensselaer, NY) and the age and sex matched SPF C57BL/6 mice were purchased from Vital River Laboratory Animal Technology Co., Ltd. (Beijing, China) via Cyagen Biosciences (Suzhou, China). Male and female GF mice were housed in a sterile isolator and the SPF control mice were housed in another isolator and fed with a NCD (Envigo, 2020SX) for analysis of the metabolic parameters. All animals were fasted for 6 h prior to sacrifice. This study complied with all relevant ethical regulations for animal testing and research. All procedures were approved by the Animal Care Committee of Shanghai Jiaotong University School of Medicine.

### Method details

#### Antibiotics (ABX) treatment

The ABX cocktail used in this study included vancomycin hydrochloride (250 mg/L; Meilunbio, China) and gentamycin sulfate (200 mg/L; Meilunbio, China). The ABX cocktail was added to the drinking water for more than two weeks and refreshed every week to eliminate the gut microbiome.

### Glucose tolerance test (GTT)

Mice were fasted for 14–16 h with free access to water and intraperitoneally injected with glucose (2 g/kg body weight in NCD mice and 1 g/kg in HFD mice) in an alternating order. Blood glucose was measured in tail vein blood before and 15, 30, 60, 90, and 120 min after glucose injection, using a glucometer and test strips (Johnson & Johnson, USA). The area under the curve (AUC) was calculated to evaluate the glucose tolerance.

### Insulin tolerance test (ITT)

Mice were fasted for 6 h and injected intraperitoneally with insulin at 1 IU/kg body weight. Blood glucose was measured in tail vein blood at the same time points with GTT. The AUCs were calculated to evaluate the insulin tolerance.

### Fecal microbiota transplantation (FMT)

All conventionally raised C57BL/6 recipient mice were pretreated with the ABX cocktail for two weeks, and ABX was withdrawn 24 h before FMT. Feces for FMT were collected from 10-week-old C57BL/6 donor mice fed a NCD or mice fed with a three-month HFD. The feces were stored in 20% glycerol at −80 °C until use. A fecal solution was introduced to the recipient mice in 200 μL of sterile PBS at a concentration of 125 mg/mL three times a week. Feces were collected before and two weeks after ABX treatment, and four weeks after FMT.

### Castration and DHT treatment experiments

Five-week-old male C57BL/6 mice were castrated (MC) or sham-operated (MS) under pentobarbital sodium anesthesia (60 mg/kg body weight). For castration, after a 5-mm incision between the penis and anus, the testes were slightly extruded out from the scrotum and excised after ligating the blood vessels. For male sham mice, an incision between the penis and anus was also performed. The testes were exposed and put back in male sham mice. The incisions were then closed with sutures. After three weeks, half of the castrated male mice were subcutaneously injected with dihydrotestosterone (DHT) (Meilunbio, China) at 2.8 mg/kg once a day for five weeks (MC + DHT). DHT was dissolved in the mixture of 90% corn oil and 10% ethanol. The mice of the other groups were injected with a mixture of 90% corn oil and 10% ethanol, as a control. Meanwhile, some of the mice in the MS, MC, and MC + DHT groups were treated with the ABX cocktail to delete the gut microbiome, and some were not. GTT and ITT were performed three and five weeks after ABX treatment, respectively.

### Cell culture

3T3-L1, C2C12, and HepG2 cell lines were obtained from American Type Culture Collection (ATCC). HepG2, 3T3-L1 and C2C12 cells were seeded into 12-well plates in growth medium DMEM with glucose (25 mmol/L) and sodium pyruvate (1 mmol/L) (Gibco11995, USA), and supplemented with 10% of fetal bovine serum (FBS) (Gibco, USA), penicillin-streptomycin solution (100 IU/mL–100 µg/mL, Gibco, USA) and L-glutamine (2 mmol/L, Gibco, USA). After 48 h’ incubation, HepG2 and 3T3-L1 cells were starved for 12 h in the growth medium, in which the FBS was replaced with 0.5% bovine serum albumin (BSA). The growth medium of C2C12 was switched to differentiation medium (DMEM, 2% horse serum, penicillin/streptomycin and L-glutamine) for 3 days before starvation. After starvation, the medium was exchanged by DMEM supplemented with 0, 50, 100 and 200 mmol/L of glutamine for 4 h for HepG2 and 3T3-L1 cells and 8 h for C2C12 cells. Cells were stimulated with or without 200 nmol/L insulin for 12 min before harvesting.

### Western blotting analysis

Tissues were homogenized with radio immunoprecipitation assay (RIPA) buffer and then incubated on ice for 30 min. The homogenates were centrifuged at 21,130 × g for 30 min at 4 °C and the supernatants were collected. The protein concentrations were quantified using the BCA Protein Assay Kit (Thermo Scientific, USA) according to the manufacturer’s protocol. A total of 20 μg of protein were fractionated using SDS-PAGE on 10% polyacrylamide gels and transferred onto PVDF membranes (Millipore, USA). After blocking with 5% BSA (Genebase, China) in TBS-T for 1 h at room temperature, the membranes were incubated with the following primary antibodies at 4 °C overnight: primary antibodies against pAkt (Cell Signalling Technology, USA), Akt (Cell Signalling Technology, USA), HSP90 (Cell Signalling Technology, USA), and anti-α-tubulin antibody (Sigma, USA). HSP90 and α-tubulin were used as internal controls. The blots were washed three times in TBS-T and then probed with the corresponding secondary antibodies (anti-rabbit/mouse IgG, HRP-linked antibody, Cell Signalling Technology, USA) for 1 h at room temperature. All the antibodies were used with 1:1000 dilution. The chemiluminescence of bands on the membrane was detected using horseradish peroxidase (HRP) substrate (Millipore, USA) and visualized using ImageQuant LAS 4000 Imaging System (GE, USA). Protein band intensities were quantified using the Image J software (Wayne Rasband, The National Institute of Mental Health, Bethesda, MD).

### Measurements of plasma parameters

Plasma was collected from mice fasted for 6 h before sacrifice. The plasma lipid levels including triglyceride (TG) and total cholesterol (TC), were measured using the commercial testing kits (Kehua, China). Free fatty acids (FFAs) were tested using a quantification colorimetric kit (BioVision, USA). Plasma insulin (Crystal Chem, USA), adiponectin (Millipore, USA), leptin (Crystal Chem, USA), LBP (Abnova, Taiwan China), L-1b, IL-6, MCP-1, TNF-α, glucagon-like peptide-1 (GLP-1) (Millipore, USA), GIP and peptide tyrosine tyrosine PYY (Crystal Chem, USA) were measured by using the corresponding enzyme-linked immunosorbent assay (ELISA) kit according to the manufacturer’s protocol. Dipeptidyl peptidase-4 (DPP-4) inhibitor (Millipore, USA) and aprotinin (Tianshuo, China) were added immediately after blood collection to inhibit the degradation of GLP-1 and other gut hormones.

### DNA extraction and 16S rRNA gene sequencing

Microbial DNA was extracted from mouse feces by using the QIAamp Fast DNA Stool mini-Kit (Qiagen, Germany) according to the manufacturer’s protocol. The concentration of bacterial DNA was measured by Nanodrop 2000. The v3-v4 regions of bacterial 16S rRNA genes were amplified at a melting temperature of 56 °C with 30 PCR cycles by using primers 341 F 5’-barcode- ACTCCTACGGGAGGCAGCAG)−3’ and 806 R 5’-GGACTACHVGGGTWTCTAAT-3’ (Supplementary Data 2). The PCR products were purified using AmpureXP beads (AGENCOURT) to remove the nonspecific products. Purified amplicons were sequenced using the Illumina MiSeq platform to obtain 300-bp paired-end reads. Raw fastq files were demultiplexed and quality-filtered using the BGI internal program. Each sample was not less than 30000 tags after being overlapped using Flash software. The clean tags were clustered into operational taxonomic units (OTUs) at a 97% identity using USEARCH. Taxonomy was assigned using the Greengenes Database. Subsequent statistical analysis was performed using R software (v3.5.1).

### Metagenomics analysis of the mouse gut microbiome

The gut microbiome of mice in the MS, MC and MC + DHT groups was investigated using a metagenomic sequencing method. Total genomic DNA was extracted from fecal samples using the QIAamp Fast DNA Stool Mini Kit (Qiagen, Germany), according to the manufacturer’s protocols. DNA was fragmented to an average size of approximately 300 bp using Covaris M220 (Gene Company Limited, China) for paired-end library construction. Paired-end sequencing was performed on Novaseq 6000 platform (Illumina Inc., San Diego, CA, USA) at Majorbio Bio-Pharm Technology Co., Ltd. (Shanghai, China) according to the manufacturer’s instructions. Adapter and low-quality reads with a length shorter than 50 bp or with a quality value score lower than 20 were discarded. The remaining reads were aligned to the Mus musculus genome using NCBI to remove the host DNA. Megahit was used to assemble the short reads. High-quality reads were mapped to the representative genes with a 95% identity using SOAPaligner and the gene abundance in each sample was evaluated. BLASTP (Version 2.2.28+) was employed for taxonomic annotations by aligning non-redundant gene catalogs against the NCBI NR database with an e-value cutoff of 1 × 10^−5^.

### Targeted amino acid profiling in human and mice

20 μL plasma was deproteinized with 80 μL of methanol containing internal standards, ALA-d3, 3 μg/mL; PHE-d5, 3 μg/mL; HIS-13C6, 1 μg/mL. The mixture was vortexed for 30 s and centrifuged at 18,407 × g for 15 min. Then 70 μL of the supernatant were freeze-dried. For the analysis, freeze-dried samples were derived using a Waters AccQ-Tag derivation kit (Waters, USA), as per manufacturer’s protocol. Boric acid buffer (70 μL, pH 8.8) were added to the freeze-dried sample and vortexed for 30 s, then 20 μL of derivation reagent were added into the mixture. After vortexing, the sample was heated in a water bath for 10 min at 55 °C.

Quantitative analysis was performed using an AB sciex ExionLC UPLC coupled to QTRAP 6500 MS system (AB Sciex, USA). The QTRAP MS data were processed by MultiQuant software (version 3.0.3, AB SCIEX, Framingham, USA). An ACQUITY UPLC C18 column (100 mm × 2.1 mm × 1.7 μm) was used for separation. Mobile phase A was water/acetonitrile/formic acid (v/v/v = 98.5:1:0.5) with 20 mmol/L ammonium formate. Mobile phase B was acetonitrile/formic acid (v/v = 98.4:1.6). Gradient elution was 1% mobile phase B to keep 1.08 min, then up to 9.1% in 10.4 min, and then to 21.2% for 5.9 min, to 59.6% in the next 0.6 min and kept for 1.2 min. The flow rate was 0.35 mL/min. The column temperature was 55 °C. Ion source parameters were set as Curtain Gas 0.241 MPa, GS1 0.276 MPa, GS2 0.276 MPa, ion source temperature 500 °C, capillary voltage 5500 V. The ion pairs and collision energy of amino acids are shown in Supplementary Data 3.

### Human study population

The subjects were recruited in our previous research, including 33 men (age, 23.27 ± 1.81 years; BMI, 20.32 ± 1.46 kg/m^2^) and 63 women (age, 23.38 ± 1.75 years; BMI, 20.23 ± 1.12 kg/m^2^) of Chinese ancestry, who were volunteers in Shanghai Jiao Tong University School of Medicine^12^. The exclusion criteria, clinical phenotypes and fasting biochemistry of these subjects were reported previously^12^. Matsuda index (10,000/square root of{[fasting glucose (mg/dL) × fasting insulin (μIU/mL)] × [mean glucose (mg/dL) × mean insulin (μIU/mL) during oral glucose tolerance test])} is highly positively correlated with the rate of whole-body glucose disposal during the euglycemic insulin clamp^34^, and was calculated in the subjects of our study. Serum testosterone was measured using a total testosterone ELISA kit (Crystalchem, USA). This study was approved by the Institutional Review Board of the Ruijin Hospital, Shanghai Jiao Tong University School of Medicine and was performed in accordance with the principles of the Helsinki Declaration II. Each participant provided a written informed consent.

### Statistics and reproducibility

Statistical analyses of metagenomics sequencing and 16S rRNA gene sequencing data were performed as described above. For the animal study, data are presented as mean ± SEM. Unpaired Student’s t-test (two-sided) was performed for the data analysis of the experiments consisting of two groups, and for the quantitative analysis of western blotting. One-way analysis of variance (ANOVA) and the post hoc test of least-significant difference (LSD) (two-tailed) were applied for the experiments consisting of more than two groups. Two-way ANOVA was used to investigate the interaction effects between sex/castration/DHT and ABX in GTT, ITT or other metabolic parameters (Supplementary Data 1). Data for Weighted Unifrac and Bray-curtis distance analysis were tested by two-tailed Wilcoxon rank-sum test. Data are expressed as mean ± SEM. Partial correlation analysis was applied to examine the correlations of glutamine, glutamate as well as their ratio with glucose metabolism indices and the serum testosterone levels with adjustment for age and BMI in men and women, respectively. Results were considered significantly different at a *P* < 0.05. Statistical analyses were performed using SAS version 9.2 (SAS Institute, USA). The statistical parameters (i.e., the n numbers) can be found in the figure legends. Each experiment was repeated independently for at least two or three times.

### Reporting summary

Further information on research design is available in the Nature Research Reporting Summary linked to this article.

## Supplementary information

Supplementary information

Description of Additional Supplementary Files

Supplementary Dataset1

Supplementary Dataset2

Supplementary Dataset3

Reporting Summary

## Data Availability

The 16S rRNA gene and metagenomic sequencing data generated in this study have been deposited in the European Molecular Biology Laboratory’s European Bioinformatics Institute (EMBL-EBI) database under accession code PRJEB41747. In 16S rRNA gene analysis, taxonomy was assigned using the Greengenes Database (https://greengenes.secondgenome.com/). In metagenomics analysis, taxonomic annotations were performed using aligning non-redundant gene catalogs against the NCBI NR database (ftp://ftp.ncbi.nlm.nih.gov/blast/db/). The source data of figures other than 16S rRNA gene and metagenomic sequencing are provided as a Source Data file with this paper. The study was approved by Chinese Ministry of Science and Technology (MOST) for the Review and Approval of Human Genetic Resources (approval number 2021BAT3969). Source data are provided with this paper.

## Source data

Source data
