# Supplementary material for: Sexual dimorphism in glucose metabolism is shaped by androgen-driven gut microbiome
Source: Nat Commun. 2021 Dec 6;12:7080. doi: 10.1038/s41467-021-27187-7 (PMC8648805; doi:10.1038/s41467-021-27187-7)
Supplement: Supplementary file 1 — Supplementary information [file 41467_2021_27187_MOESM1_ESM.pdf]

Supplementary Materials for

Sexual dimorphism in glucose metabolism is shaped by androgen-driven gut microbiome

Aibo Gao, Junlei Su, Ruixin Liu, Shaoqian Zhao, Wen Li, Xiaoqiang Xu, Danjie Li, Juan Shi, Bin Gu, Juan Zhang, Qi Li, Xiaolin Wang, Yifei Zhang, Yu Xu, Jieli Lu, Guang Ning, Jie Hong, Yufang Bi, Weiqiong Gu, Jiqiu Wang, Weiqing Wang

Correspondence to:

weiqionggu@163.com

wangjq@shsmu.edu.cn

wqingw61@163.com

The Supplementary Information of this study consist of:

-5 Supplementary Figures

-3 Supplementary Datasets

Supplementary Text

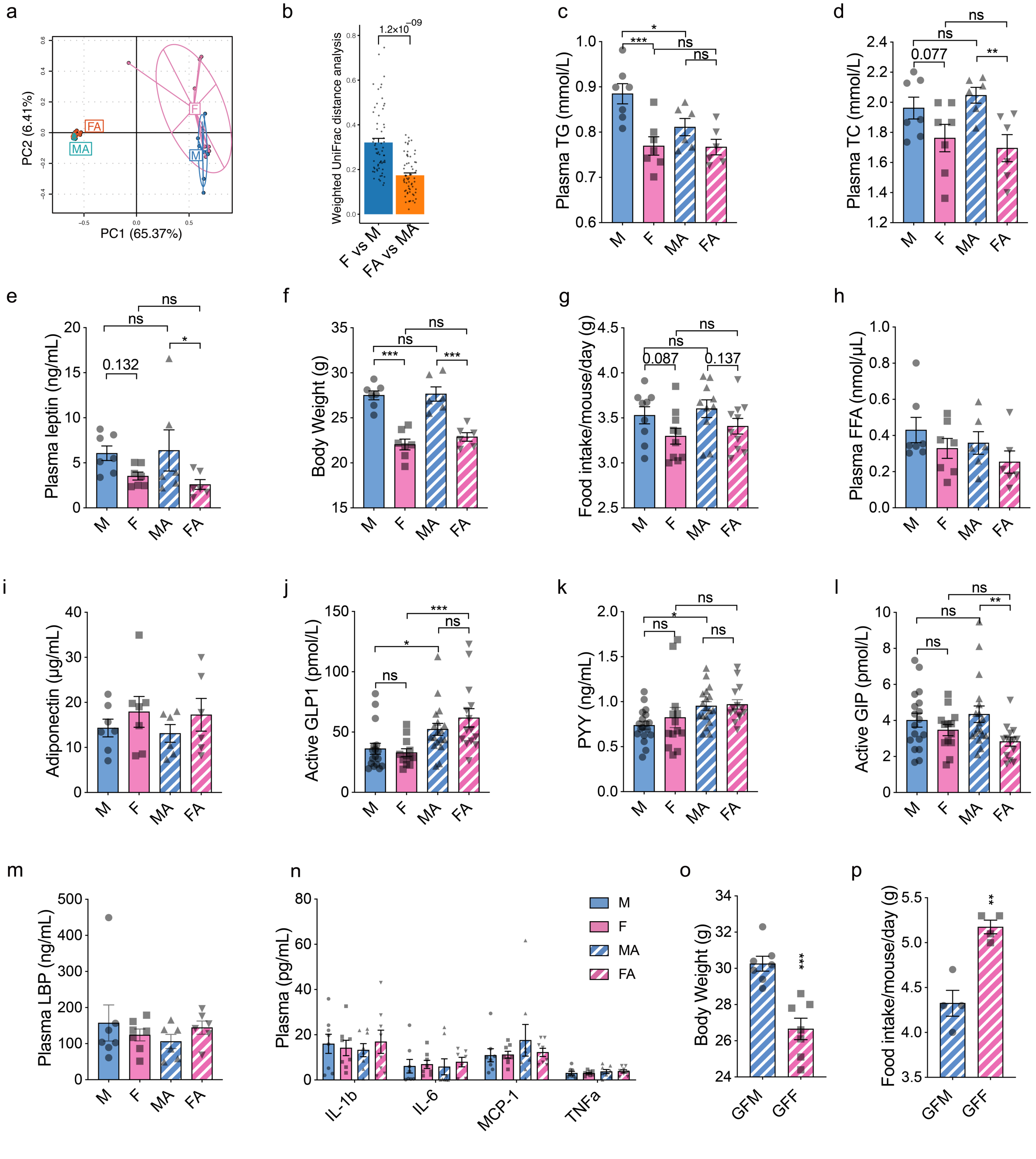

Supplementary Fig. 1. Metabolic phenotypes of normal chow diet (NCD) fed male and female mice without or with antibiotics (ABX) treatment.

a Principal component analysis (PCA) of the gut microbiome based on operational taxonomic units (OTUs) of the indicated groups (mice, n = 8 per group). **b** Weighted UniFrac distance analysis of the gut microbiome based on the OTUs of the indicated groups (mice, n = 8 per group). **c-e** Fasting plasma concentrations of triglyceride (TG) (c) (for M VS F, $P = 3.6 \times 10^{-4}$ and for M VS MA, $P = 0.017$) , total cholesterol (TC) (d) (for M VS F, $P = 0.077$ and for MA VS FA, $P = 0.006$), and leptin (e) (for M VS F, $P = 0.132$ and for MA VS FA, $P = 0.042$) in the indicated groups (mice, n = 7 for M and F; n = 6 for MA and FA). **f, g** Body weight (f) (for M VS F, $P = 9.8 \times 10^{-7}$ and for MA VS FA, $P = 1.9 \times 10^{-5}$) (mice, n = 7 for M and F; n = 6 for MA and FA) and food intake per mouse per day (g) (for M VS F, $P = 0.087$ and for MA VS FA, $P = 0.137$) (cages, n = 9 for M; cages, n = 10 for F, MA, and FA) in the indicated groups. **h-l** Fasting plasma concentrations of free fatty acids (FFAs) (h) (mice, n = 7 for M and F; n = 6 for MA and FA), adiponectin (i) (mice, n = 7 for M and F; n = 6 for MA and FA) , active glucagon-like peptide-1 (GLP-1) (j) (for M VS MA, $P = 0.026$ and for F VS FA, $P = 8.8 \times 10^{-4}$) (mice, n = 17 for M; n = 12 for F; n = 18 for MA ; n = 14 for FA), peptide tyrosine tyrosine (PYY) (k) (for M VS MA, $P = 0.013$) (mice, n = 18 for M; n = 13 for F; n = 18 for MA ; n = 14 for FA) , and glucose-dependent insulinotropic polypeptide (GIP) (l) (for MA VS FA, $P = 0.007$) (mice, n = 18 for M; n = 13 for F; n = 18 for MA ; n = 14 for FA) in the indicated groups. **m, n** Plasma concentrations of lipopolysaccharide binding protein (LBP) (m) (mice, n = 7 for M and F; n = 6 for MA and FA) and interleukin-1b (IL-1b), interleukin-6 (IL-6), monocyte chemotactic protein 1 (MCP1), and tumor necrosis factor alpha (TNFα) (n) (mice, n = 8 per group) in male and female mice without or with ABX treatment. **o, p** Body weight (o) (for GFM VS GFF, $P = 3.1 \times 10^{-4}$) (mice, n = 7 per group) and food intake (p) (for GFM VS GFF, $P = 0.002$) (cages, n = 4 per group) in male and female GF mice. M, male; F, female; MA, male mice after ABX treatment; FA, female mice after ABX treatment; GFM, male germ-free

mice. GFF, female germ-free mice. *$P < 0.05$, **$P < 0.01$, ***$P < 0.001$; ns, not significant. Data for Weighted Unifrac distance analysis in supplementary Fig 1b were tested by two-tailed Wilcoxon rank-sum test. One-way ANOVA and the post hoc test of least-significant difference (LSD) (two-sided) was applied to analyze the data in Supplementary Fig1 c to n. Unpaired Student's t-test (two sided) was performed for Supplementary Fig1 o to p. Data are expressed as mean ± SEM. Source data are provided as a Source Data file.

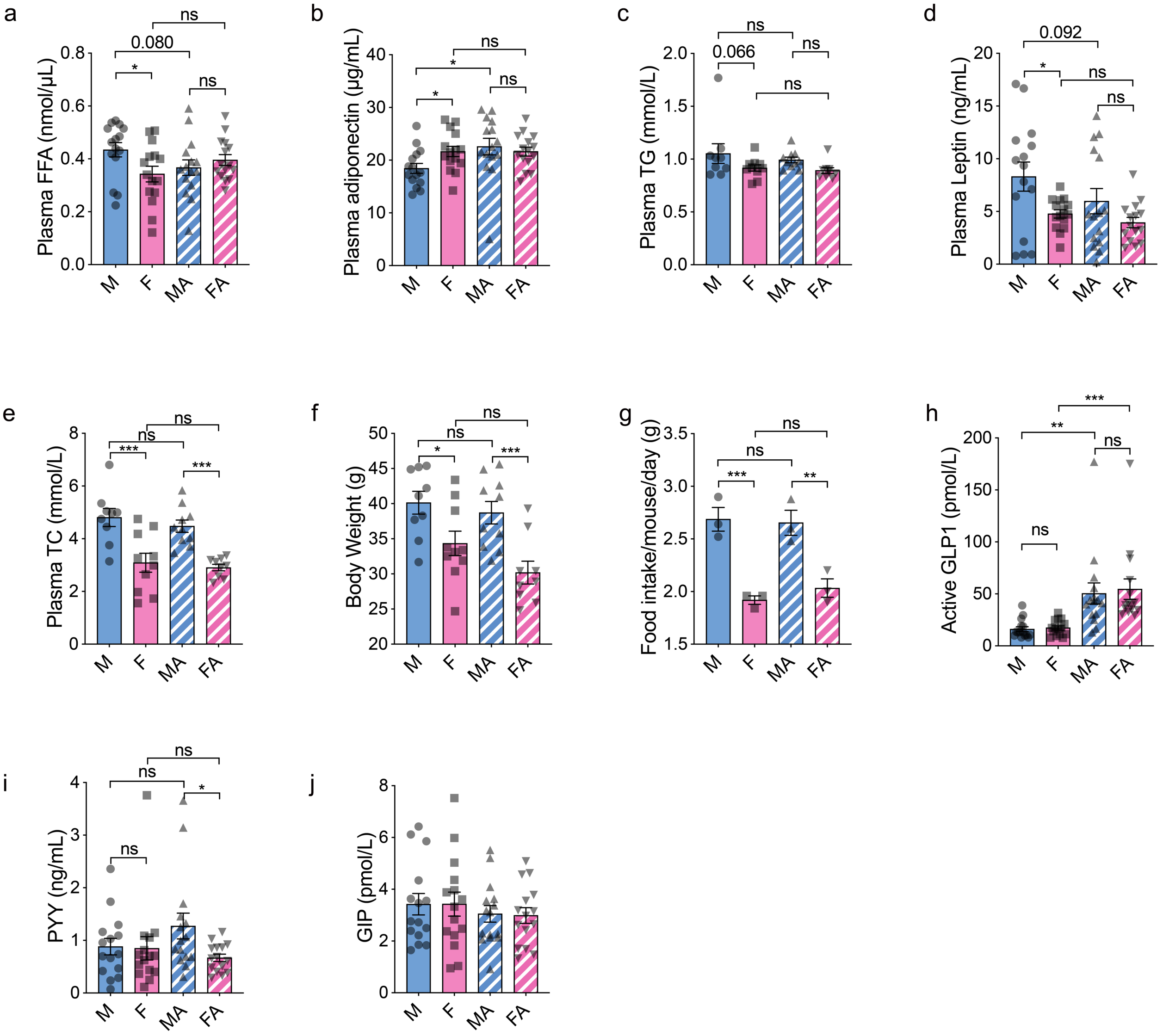

Supplementary Fig.2. Metabolic phenotypes of high-fat diet (HFD) fed male and female mice without or with ABX treatment.

a-e, Fasting plasma concentrations of FFA (a) (for M VS F, $P = 0.019$; for M VS MA, $P = 0.080$) (mice, n = 15 per group), adiponectin (b) (for M VS F, $P = 0.047$; for M VS MA, $P = 0.011$) (mice, n = 15 per group), TG (c) (for M VS F, $P = 0.066$) (mice, n = 9 for M; n = 10 for F and MA; n = 9 for FA), leptin (d) (for M VS F, $P = 0.012$; for M VS MA, $P = 0.092$) (mice, n = 15 per group), and TC (e) (for M VS F, $P = 1.5 \times 10^{-4}$; for MA VS MA, $P = 4.3 \times 10^{-4}$) (mice, n = 9 for M; n =

10 for F and MA; n = 9 for FA) in HFD fed male and female mice without or with ABX treatment. **f** Body weight (for M VS F, $P = 0.018$; for MA VS MA, $P = 8.8 \times 10^{-4}$) (mice, n = 9 for M; n = 10 for F and MA; n = 9 for FA). **g** Food intake per mouse per day (for M VS F, $P = 4.5 \times 10^{-4}$; for MA VS MA, $P = 0.002$) (cages, n = 3 per group). **h-j** Fasting plasma concentrations of active GLP-1 (h) (for M VS MA, $P = 0.001$; for F VS FA, $P = 6.0 \times 10^{-4}$), PYY (i) (for MA VS FA, $P = 0.025$, and GIP (j) in the indicated groups (h-j, mice, n = 15 per group). FFAs, free fatty acids, TG, triglyceride; TC, total cholesterol; GLP-1, glucagon-like peptide-1; PYY, peptide tyrosine tyrosine; GIP, glucose-dependent insulinotropic polypeptide. M, male; F, female; MA, male mice after ABX treatment; FA, female mice after ABX treatment. *$P < 0.05$, **$P < 0.01$, ***$P < 0.001$; ns, not significant. One-way ANOVA and the post hoc test of LSD (two-sided) was applied to analyze the data in Supplementary Fig2. Data are expressed as mean ± SEM. Source data are provided as a Source Data file.

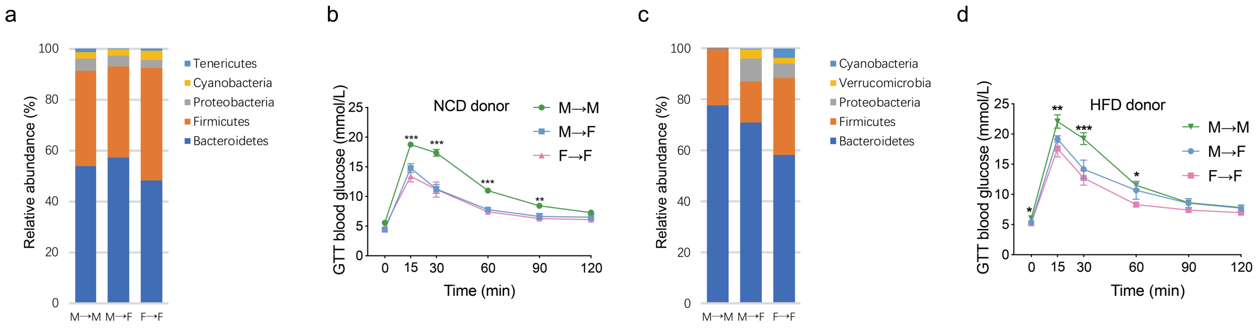

Supplementary Fig. 3. Fecal microbiota transplantation (FMT) alters the gut microbiome. **a** The relative abundance of the five most abundant microbial phyla in M→M, M→F, and F→F groups (mice, n = 11 for M→M and F→F; n = 9 for M→F). The donors were fed a NCD. **b** GTT in M→M, M→F, and F→F groups (for M→M VS F→F, $P = 2.8 \times 10^{-5}$ at 15 min; $P = 3.1 \times 10^{-5}$ at 30 min; $P = 1.2 \times 10^{-6}$ at 60 min; $P = 0.002$ at 90 min) (mice, n = 11 for M→M; n = 7 for F→F; n = 11 for M→F). The donors were fed a NCD. **c** The relative abundance of the five most abundant microbial phyla in M→M, M→F, and F→F groups (mice, n = 13 for M→M and F→F; n = 8 for M→F). The donors were fed a HFD. **d** GTT in M→M, M→F, and F→F groups (for M→M VS F→F, $P = 0.027$ at 0 min; $P = 0.006$ at 15 min; $P = 6.0 \times 10^{-4}$ at 30 min; $P = 0.023$ at 60 min) (mice, n = 13 for M→M; n = 12 for F→F and M→F). The donors were fed a HFD. GTT, glucose tolerance test; NCD, normal chow diet; HFD, high fat diet. M→M, male feces transferred to male recipients; M→F, male feces transferred to female recipients; F→F, female feces transferred to female recipients. For Supplementary Fig3 b and d, M→M VS F→F, $^{*}P < 0.05$, $^{**}P < 0.01$, $^{***}P < 0.001$. One-way ANOVA and the post hoc test of LSD (two-sided) was applied to analyze the data in Supplementary Fig3 b and d. Data are expressed as mean ± SEM. Source data are provided as a Source Data file.

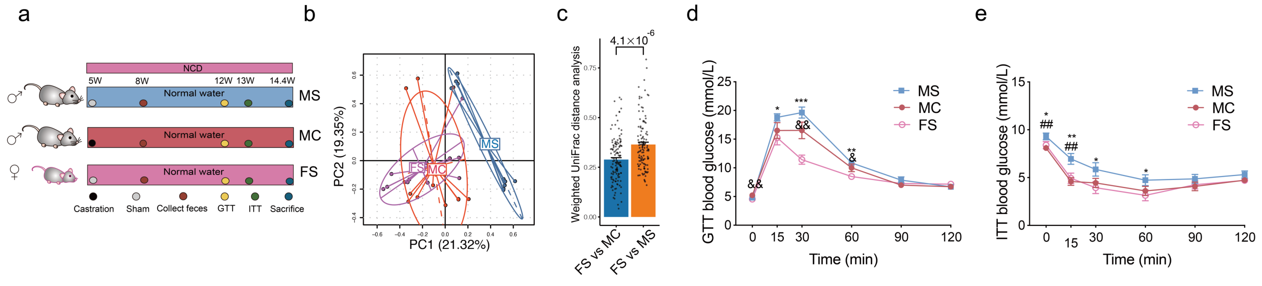

Supplementary Fig. 4. Androgen depletion alters the gut microbiome and improves glucose metabolism

a Schematic diagram of male mice subjected to castration and sham operation. **b** PCA of the microbiome of MS, MC, and FS groups based on the OTUs (mice, n = 10 for MS; n = 11 for MC; n = 12 for FS). **c** Weighted UniFrac distance analysis of the gut microbiome in the indicated groups (mice, n = 10 for MS; n = 11 for MC; n = 12 for FS). **d, e** GTT (d) (for MC VS FS, $P = 0.008$ at 0 min; for MS VS FS, $P = 0.041$ at 15 min; for MS VS FS, $P = 1.3 \times 10^{-5}$ and MC VS FS, $P = 0.003$ at 30 min; for MS VS FS, $P = 0.003$ and MC VS FS, $P = 0.041$ at 60 min) (mice, n = 11 for MS; n = 12 for MC and FS) and ITT (e) (for MS VS MC, $P = 0.009$ and for MS VS FS, $P = 0.043$ at 0 min; for MS VS MC, $P = 0.003$ and for MS VS FS, $P = 0.009$ at 15 min; for MS VS FS, $P = 0.032$ at 30 min; for MS VS FS, $P = 0.048$ at 60 min) (mice, n = 11 for MS; n = 12 for MC and FS) of the indicated groups. For d and e, MS vs FS, $^{*}P < 0.05$, $^{**}P < 0.01$, $^{***}P < 0.001$; MS vs MC, $\#P < 0.05$, $\#\#P < 0.01$; MC vs FS, $\&P < 0.05$, $\&\&P < 0.01$. NCD, normal chow diet; GTT, glucose tolerance test; ITT, insulin tolerance test. MS, male sham mice; MC, male castrated mice; FS, female sham mice. Data for Weighted Unifrac distance analysis in supplementary Fig 4c were tested by two-tailed Wilcoxon rank-sum test. One-way ANOVA and the post hoc test of LSD (two-

sided) was applied to analyze the data in Supplementary Fig4 d to e. Data are expressed as mean ± SEM. Source data are provided as a Source Data file.

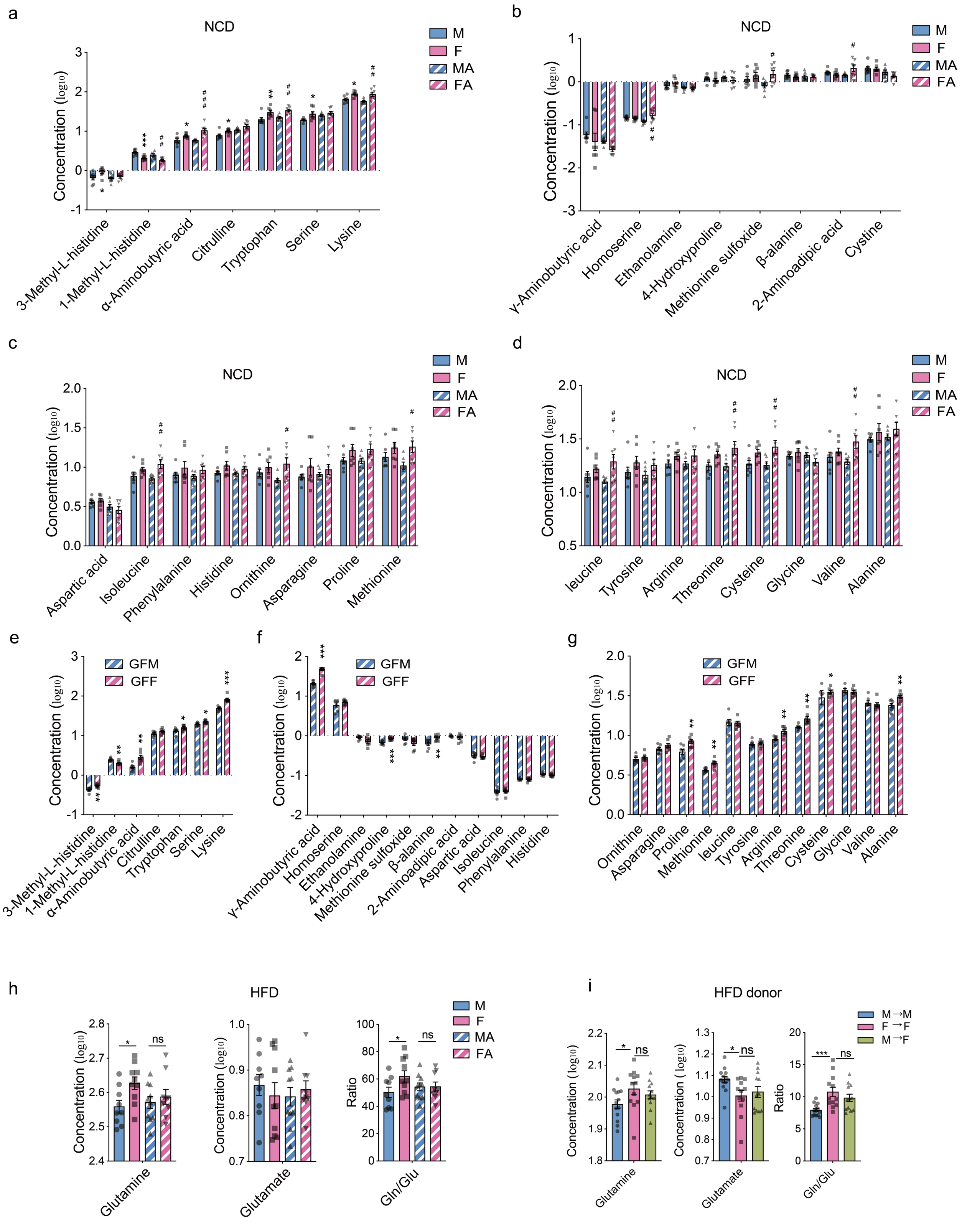
a
NCD
M
F
MA
FA
Concentration (log10)
3-Methyl-L-histidine
1-Methyl-L-histidine
α-Aminobutyric acid
Citrulline
Tryptophan
Serine
Lysine
b
NCD
γ-Aminobutyric acid
Homoserine
Ethanolamine
4-Hydroxyproline
Methionine sulfoxide
β-alanine
2-Aminoadipic acid
Cystine
c
NCD
Aspartic acid
Isoleucine
Phenylalanine
Histidine
Ornithine
Asparagine
Proline
Methionine
d
NCD
leucine
Tyrosine
Arginine
Threonine
Cysteine
Glycine
Valine
Alanine
e
GFM
GFF
f
g
h
HFD
Glutamine
Glutamate
Gln/Glu
Ratio
i
HFD donor
M→M
F→F
M→F

Supplementary Fig.5. Plasma concentrations of amino acids in male and female mice.

a-d Fasting plasma concentrations of amino acids in male and female mice fed a NCD without or with ABX treatment (in 3-Methyl-L-histidine, for M VS F, $P = 0.019$; in 1-Methyl-L-histidine, for M VS F, $P = 7.7 \times 10^{-4}$ and for MA VS FA, $P = 0.002$; in α-Aminobutyric acid, for M VS F, $P = 0.033$ and for MA VS FA, $P = 1.0 \times 10^{-4}$; in Citrulline, for M VS F, $P = 0.025$; in Tryptophan, for M VS F, $P = 0.004$ and for MA VS FA, $P = 0.005$; in Serine, for M VS F, $P = 0.010$; in Lysine, for M VS F, $P = 0.012$ and for MA VS FA, $P = 0.002$; in Homoserine, for MA VS FA, $P = 0.003$; in Methionine sulfoxide, for MA VS FA, $P = 0.012$; in 2-Aminoadipic acid, for MA VS FA, $P = 0.018$; in Isoleucine, for MA VS FA, $P = 0.005$; in Ornithine, for MA VS FA, $P = 0.013$, in Methionine, for MA VS FA, $P = 0.012$; in leucine, for MA VS FA, $P = 0.010$; in Threonine, for MA VS FA, $P = 0.007$; in Cysteine, for MA VS FA, $P = 0.007$; in Valine, for MA VS FA, $P = 0.007$) (mice, n = 7 per group). For a-d, M vs F, *$P < 0.05$, **$P < 0.01$, ***$P < 0.001$; MA vs FA, #$P < 0.05$, ##$P < 0.01$, ###$P < 0.001$. **e-g** Fasting plasma concentrations of the indicated amino acids in GF mice (for GFM VS GFF, in 3-Methyl-L-histidine, $P = 0.002$; in 1-Methyl-L-histidine, $P = 0.003$; in α-Aminobutyric acid, $P = 0.001$; in Tryptophan, $P = 0.036$; in Serine, $P = 0.015$; in Lysine, $P = 3.2 \times 10^{-5}$; in γ-Aminobutyric acid, $P = 2.5 \times 10^{-7}$; in 4-Hydroxyproline, $P = 7.4 \times 10^{-4}$; in β-alanine, $P = 0.009$; in Proline, $P = 0.006$; in Methionine, $P = 0.002$; in Arginine, $P = 0.004$; in Threonine, $P = 0.006$; in Cysteine, $P = 0.011$; in Alanine, $P = 0.009$) (mice, n = 7 per group). **h** Fasting plasma concentrations of glutamine, glutamate, and Gln/Glu ratio in HFD fed male and female mice without or with ABX treatment (in glutamine, for M VS F, $P = 0.015$; in Gln/Glu, for M VS F, $P = 0.027$) (mice, n = 9 for M, n = 10 for F and MA; n = 9 for FA). **i** Fasting plasma concentrations of glutamine, glutamate, and Gln/Glu ratio in M→M, F→F, and M→F groups. The donors were fed a HFD (in glutamine, for M→M VS F→F, $P = 0.029$; in glutamate, for M→M

VS F→F, $P = 0.021$; in Gln/Glu, for M→M VS F→F, $P = 9.1 \times 10^{-4}$) (mice, n = 13 for M→M; n = 12 for M→F and F→F). The concentration of amino acids is calculated as $\log_{10}$ and the ratio of glutamine to glutamate is calculated directly by the glutamine concentration to the glutamate concentration. NCD, normal chow diet; HFD, high fat diet. M, male; F, female; MA, male mice after ABX treatment; FA, female mice after ABX treatment. GFM, male germ free mice; GFF, female germ free mice. M→M, male feces transferred to male recipients; F→F, female feces transferred to female recipients; M→F, male feces transferred to female recipients. $^{*}P < 0.05$, $^{**}P < 0.01$, $^{***}P < 0.001$; ns, not significant. One-way ANOVA and the post hoc test of LSD (two-sided) was applied to analyze the data in Supplementary Fig5. Data are expressed as mean ± SEM. Source data are provided as a Source Data file.
