# Supplementary material for: Sexual dimorphism in glucose metabolism is shaped by androgen-driven gut microbiome
Source: Nat Commun. 2021 Dec 6;12:7080. doi: 10.1038/s41467-021-27187-7 (PMC8648805; doi:10.1038/s41467-021-27187-7)
Supplement: Supplementary file 2 — Description of Additional Supplementary Files [file 41467_2021_27187_MOESM2_ESM.docx]

Description of Additional Supplementary Files

Title: Supplementary Data 1

Description: Two-way ANOVA analyses in different figures.

Title: Supplementary Data 2

Description: The primers used in this study.

Title: Supplementary Data 3

Description: Ion pairs and collision energy of amino acids.
