# Supplementary material for: Sexual dimorphism in glucose metabolism is shaped by androgen-driven gut microbiome
Source: Nat Commun. 2021 Dec 6;12:7080. doi: 10.1038/s41467-021-27187-7 (PMC8648805; doi:10.1038/s41467-021-27187-7)
Supplement: Supplementary file 6 — Reporting Summary [file 41467_2021_27187_MOESM6_ESM.pdf]

nature portfolio

Corresponding author(s): Weiqiong Gu, Jiqiu Wang, Weiqing Wang

Last updated by author(s): Sep 1, 2021

# Reporting Summary

Nature Portfolio wishes to improve the reproducibility of the work that we publish. This form provides structure for consistency and transparency in reporting. For further information on Nature Portfolio policies, see our Editorial Policies and the Editorial Policy Checklist.

## Statistics

For all statistical analyses, confirm that the following items are present in the figure legend, table legend, main text, or Methods section.

| n/a | Confirmed | |
|---|---|---|
| ☐ | ☒ | The exact sample size (*n*) for each experimental group/condition, given as a discrete number and unit of measurement |
| ☐ | ☒ | A statement on whether measurements were taken from distinct samples or whether the same sample was measured repeatedly |
| ☐ | ☒ | The statistical test(s) used AND whether they are one- or two-sided<br>*Only common tests should be described solely by name; describe more complex techniques in the Methods section.* |
| ☐ | ☒ | A description of all covariates tested |
| ☐ | ☒ | A description of any assumptions or corrections, such as tests of normality and adjustment for multiple comparisons |
| ☐ | ☒ | A full description of the statistical parameters including central tendency (e.g. means) or other basic estimates (e.g. regression coefficient) AND variation (e.g. standard deviation) or associated estimates of uncertainty (e.g. confidence intervals) |
| ☐ | ☒ | For null hypothesis testing, the test statistic (e.g. *F*, *t*, *r*) with confidence intervals, effect sizes, degrees of freedom and *P* value noted<br>*Give P values as exact values whenever suitable.* |
| ☒ | ☐ | For Bayesian analysis, information on the choice of priors and Markov chain Monte Carlo settings |
| ☒ | ☐ | For hierarchical and complex designs, identification of the appropriate level for tests and full reporting of outcomes |
| ☒ | ☐ | Estimates of effect sizes (e.g. Cohen's *d*, Pearson's *r*), indicating how they were calculated |

*Our web collection on statistics for biologists contains articles on many of the points above.*

## Software and code

Policy information about availability of computer code

| | |
|---|---|
| Data collection | The Immunoblotting bands were visualized using ImageQuant LAS 4000 Imaging System (GE, USA). Protein band intensities were quantified using the Image J software (Wayne Rasband, The National Institute of Mental Health, Bethesda, MD). Quantitative analysis of amino acids was performed using an AB sciex ExionLC UPLC coupled to QTRAP 6500 MS system (AB Sciex, USA). The QTRAP MS data were processed by MultiQuant software (version 3.0.3, AB SCIEX, Framingham, U.S.A.). 16S rRNA gene sequencing was performed on illumina Miseq (Illumina Inc., San Diego, CA, USA). Metagenomics sequencing was performed on Novaseq 6000 platform (Illumina Inc., San Diego, CA, USA). |
| Data analysis | Statistical analyses were performed using SAS version 9.2 or R 3.5.1. |

For manuscripts utilizing custom algorithms or software that are central to the research but not yet described in published literature, software must be made available to editors and reviewers. We strongly encourage code deposition in a community repository (e.g. GitHub). See the Nature Portfolio guidelines for submitting code & software for further information.

## Data

Policy information about availability of data

All manuscripts must include a data availability statement. This statement should provide the following information, where applicable:

- Accession codes, unique identifiers, or web links for publicly available datasets
- A description of any restrictions on data availability
- For clinical datasets or third party data, please ensure that the statement adheres to our policy

The 16S rRNA genes and metagenomic sequencing data generated in this study have been deposited in the European Molecular Biology Laboratory's European Bioinformatics Institute (EMBL-EBI) database under accession code PRJEB41747. In 16S rRNA gene analysis, taxonomy was assigned using the Greengenes Database (https://greengenes.secondgenome.com/). In metagenomics analysis, taxonomic annotations were performed using aligning non-redundant gene catalogs against

the NCBI NR database (ftp://ftp.ncbi.nlm.nih.gov/blast/db/). Source data are provided with this paper.

# Field-specific reporting

Please select the one below that is the best fit for your research. If you are not sure, read the appropriate sections before making your selection.

☒ Life sciences ☐ Behavioural & social sciences ☐ Ecological, evolutionary & environmental sciences

For a reference copy of the document with all sections, see nature.com/documents/nr-reporting-summary-flat.pdf

# Life sciences study design

All studies must disclose on these points even when the disclosure is negative.

| | |
|---|---|
| Sample size | No statistical methods were used to predetermine sample size. Sample size was selected based on previous experiments in the prior publications of our lab (1) and that of others using comparable methods (2,3).<br>1. Liu R, et al. Gut microbiome and serum metabolome alterations in obesity and after weight-loss intervention. Nature Medicine 23, 859-868 (2017).<br>2. Suárez-Zamorano N, et al. Microbiota depletion promotes browning of white adipose tissue and reduces obesity. Nature Medicine 21, 1497-1501 (2015).<br>3. Zarrinpar A, et al. Antibiotic-induced microbiome depletion alters metabolic homeostasis by affecting gut signaling and colonic metabolism. Nat Commun 9, (2018). |
| Data exclusions | Exclusion criteria is pre-established before the experiments. If done usually due to samples that deemed to be outliers or animals in abnormal condition. If the individual value is 3 times the interquartile range (IQR) higher than the third quartile or 3 times IQR lower than the first quartile of the box plot, it is generally considered as an outlier.<br>1. One outlying sample was excluded in the insulin tolerance test (GTT) and the corresponding area under curve (AUC) in the ABX treatment experiment in Fig1 g and h.<br>2. One outlying sample was excluded in the glucose tolerance test (ITT) and the corresponding AUC in the ABX treatment experiment in Fig2 f and g.<br>3. Two outlying samples were excluded in the plasma GLP-1 levels in the ABX treated experiment in Supplementary Fig1.<br>4. One mice died before sacrifice in ABX treated experiment and its data in the supplementary fig2 was not collected.<br>5. 16S rRNA gene and metagenome sequencing were not analyzed if failed to collect the feces or build the library.<br>6. Plasma insulin levels were excluded if the mice were injected with insulin before sacrifice. |
| Replication | Each experiment was repeated independently at least for two or three times. |
| Randomization | No randomization was used in this study, and the acclimatized mice were allocated to groups on the basis of their body weights in each sex to ensure equal starting points. |
| Blinding | The study and experiments were not blinded due to obvious differences or different treatments between groups. However, two investigators performed the experiments and independently analyzed the data. |

# Reporting for specific materials, systems and methods

We require information from authors about some types of materials, experimental systems and methods used in many studies. Here, indicate whether each material, system or method listed is relevant to your study. If you are not sure if a list item applies to your research, read the appropriate section before selecting a response.

## Materials & experimental systems

| n/a | Involved in the study | |
|---|---|---|
| ☐ | ☒ | Antibodies |
| ☐ | ☒ | Eukaryotic cell lines |
| ☒ | ☐ | Palaeontology and archaeology |
| ☐ | ☒ | Animals and other organisms |
| ☐ | ☒ | Human research participants |
| ☒ | ☐ | Clinical data |
| ☒ | ☐ | Dual use research of concern |

## Methods

| n/a | Involved in the study | |
|---|---|---|
| ☒ | ☐ | ChIP-seq |
| ☒ | ☐ | Flow cytometry |
| ☒ | ☐ | MRI-based neuroimaging |

## Antibodies

| | |
|---|---|
| Antibodies used | 1. Rabbit-anti-Akt (pan) (Cell Signalling Technology, clone C67E7, Cat# 4691) (1:1000 dilluted)<br>2. Rabbit-anti-Phospho-Akt (Ser473) (Cell Signalling Technology, clone D9E, Cat# 4060) (1:1000 dilluted)<br>3. Rabbit-anti-HSP90 (Cell Signalling Technology, Clone C45G5, Cat# 4877) (1:1000 dilluted)<br>4. Mouse-anti-α-Tubulin antibody, (Sigma, clone AA13, Cat# T8203) (1:1000 dilluted) (1:1000 dilluted) |

| | |
|---|---|
| | 5. Goat-anti-rabbit IgG, HRP-linked Antibody (Cell Signalling Technology, Cat# 7074) (1:1000 dilluted)<br>6. Goat-anti-mouse IgG, HRP-linked Antibody (Cell Signalling Technology, Cat# 7076) (1:1000 dilluted) |
| Validation | Validation data about the antibodies purchased from commercial vendors are available on the manufacturer's website and/or datasheets sent to us. And the antibodies are widely used in the scientific community.<br>1. Rabbit-anti-Akt (pan) (Cell Signalling Technology, clone C67E7, Cat# 4691) (1:1000 dilluted) https://www.cellsignal.cn/products/primary-antibodies/akt-pan-c67e7-rabbit-mab/4691?site-search-type=Products&N=4294956287&Ntt=4691&fromPage=plp&_requestid=1672739<br>2. Rabbit-anti-Phospho-Akt (Ser473) (Cell Signalling Technology, clone D9E, Cat# 4060) (1:1000 dilluted) https://www.cellsignal.cn/products/primary-antibodies/phospho-akt-ser473-d9e-xp-rabbit-mab/4060?site-search-type=Products&N=4294956287&Ntt=4060&fromPage=plp&_requestid=1672786<br>3. Rabbit-anti-HSP90 (Cell Signalling Technology, Clone C45G5, Cat# 4877) (1:1000 dilluted) https://www.cellsignal.cn/products/primary-antibodies/hsp90-c45g5-rabbit-mab/4877?site-search-type=Products&N=4294956287&Ntt=4877&fromPage=plp&_requestid=1673797<br>4. Mouse-anti-α-Tubulin antibody, (Sigma, clone AA13, Cat# T8203) (1:1000 dilluted) (1:1000 dilluted) https://www.sigmaaldrich.cn/CN/zh/product/sigma/t8203?context=product<br>5. Goat-anti-rabbit IgG, HRP-linked Antibody (Cell Signalling Technology, Cat# 7074) (1:1000 dilluted) https://www.cellsignal.cn/products/secondary-antibodies/anti-rabbit-igg-hrp-linked-antibody/7074?site-search-type=Products&N=4294956287&Ntt=7074&fromPage=plp&_requestid=1673970<br>6. Goat-anti-mouse IgG, HRP-linked Antibody (Cell Signalling Technology, Cat# 7076) (1:1000 dilluted) https://www.cellsignal.cn/products/secondary-antibodies/anti-mouse-igg-hrp-linked-antibody/7076?site-search-type=Products&N=4294956287&Ntt=7076&fromPage=plp&_requestid=1674071 |

## Eukaryotic cell lines

Policy information about cell lines

| | |
|---|---|
| Cell line source(s) | 3T3-L1 cell line, C2C12 cell line, and HepG2 cell line were obtained from American Type Culture Collection (ATCC). |
| Authentication | 3T3-L1, C2C12 and HepG2 cell lines were authenticated by post-freeze viability, growth properties, morphology, as well as PCR assays with species-specific primers. |
| Mycoplasma contamination | Cells have been routinely tested for mycoplasma and they are negative. |
| Commonly misidentified lines<br>(See ICLAC register) | No commonly misidentified cell lines were used. |

## Animals and other organisms

Policy information about studies involving animals; ARRIVE guidelines recommended for reporting animal research

| | |
|---|---|
| Laboratory animals | C57BL/6 specific pathogen free (SPF) male and female mice were 8 weeks at the beginning of antibiotics treatment experiment, 3 weeks at the beginning of fecal microbiota transplantation experiment, and were 5 weeks when underwent the castration surgery. C57BL/6 SPF mice and germ free (GF) mice were used in the study began at 8 weeks. |
| Wild animals | The study did not involve wild animals. |
| Field-collected samples | The study did not involve samples collected from the field. |
| Ethics oversight | All procedures were approved by the Animal Care Committee of Shanghai Jiaotong University School of Medicine. |

Note that full information on the approval of the study protocol must also be provided in the manuscript.

## Human research participants

Policy information about studies involving human research participants

| | |
|---|---|
| Population characteristics | The subjects were recruited in our previous research, including 33 men (age, 23.27 ± 1.81 years; BMI, 20.32 ± 1.46 kg/m2) and 63 women (age, 23.38 ± 1.75 years; BMI, 20.23 ± 1.12 kg/m2) of Chinese ancestry, who were volunteers in Shanghai Jiao Tong University School of Medicine. |
| Recruitment | Healthy controls were recruited from volunteers of Shanghai Jiao Tong University School of Medicine. The exclusion criteria for controls were: hypertension; impaired glucose regulation; diabetes mellitus; history of obesity, gastrointes<br>tinal disease and gastrointestinal surgery within 5 years before recruitment; and abnormal liver and kidney function, and blood lipid levels. |
| Ethics oversight | This study was approved by the Institutional Review Board of the Ruijin Hospital, Shanghai Jiao Tong University School of Medicine and was performed in accordance with the principle of the Helsinki Declaration II. Each participant provided a written informed consent. |

Note that full information on the approval of the study protocol must also be provided in the manuscript.
